# Late-Stage Functionalization through Click Chemistry Provides GLUT5-Targeting Glycoconjugate as a Potential PET Imaging Probe

**DOI:** 10.3390/ijms24010173

**Published:** 2022-12-22

**Authors:** Adelina Oronova, Marina Tanasova

**Affiliations:** 1 Chemistry Department, Michigan Technological University, Houghton, MI 49931, USA; 2 Health Research Institute, Michigan Technological University, Houghton, MI 49931, USA

**Keywords:** PET imaging, click chemistry, sugar transport, fructose transport, cancer imaging, GLUTs, glycoconjugates, sugar–disease connection

## Abstract

The targeting of facilitative sugar transporters (GLUTs) has been utilized in the development of tools for diagnostics and therapy. The interest in this area is promoted by the phenomenon of alterations in cellular metabolic processes that are linked to multitudes of metabolic disorders and diseases. However, nonspecific targeting (e.g., glucose-transporting GLUTs) leads to a lack of disease detection efficiency. Among GLUTs, GLUT5 stands out as a prominent target for developing specific molecular tools due to its association with metabolic diseases, including cancer. This work reports a non-radiolabeled fluoride (^19^F) coumarin-based glycoconjugate of 2,5-anhydro-D-mannitol as a potential PET imaging probe that targets the GLUT5 transporter. Inherent fluorescent properties of the coumarin fluorophore allowed us to establish the probe’s uptake efficiency and GLUT5-specificity in a GLUT5-positive breast cell line using fluorescence detection techniques. The click chemistry approach employed in the design of the probe enables late-stage functionalization, an essential requirement for obtaining the radiolabeled analog of the probe for future in vivo cancer imaging applications. The high affinity of the probe to GLUT5 allowed for the effective uptake in nutrition-rich media.

## 1. Introduction

The development of small-molecule probes constitutes a central part of chemical biology research that provides tools for exploring mechanisms of biological pathways. These molecular tools aid in the assessment of emerging therapeutic targets and the development of biomedical agents. Among prominent biological pathways directly associated with diseases are membrane sugar transporters that transport various carbohydrates in and out of the cell. Unlike sodium-coupled glucose transporter, facilitative sugar transporters—GLUTs—translocate glucose, fructose, galactose, and other sugars based on the gradient. Recently, GLUTs have gained interest due to the growing evidence of links between the activity of these transporters and metabolic disorders, including diabetes, obesity, and cancer [1,2].

A positive correlation between high sugar uptake and cancer identified over the years has led to the development of strategies to target sugar uptake for cancer diagnosis and therapy [3,4,5]. A significant impact on advancing disease detection has been made by the development of radiolabeled glucose analogs and their use in the clinic for cancer diagnosis. The ^18^F-labeled analog of 2-fluoro-2-deoxy-D-glucose (2-^18^F-FDG) has been effectively used as a reflector of enhanced glucose uptake and predictor of tumorigenesis and tumor aggressiveness [5]. 2-^18^F-FDG was established to be readily internalized by the cell through glucose GLUTs and further phosphorylation, promoting continuous gradient-dependent uptake. Numerous efforts were also directed to develop radiotracers that would reflect regional glucose content and cellular uptake independent of phosphorylation that would still provide gradient-dependent uptake. [^123^I]-6-deoxy-6-iodo-D-glucose (6-DIG) [6] was shown to pass into cells through glucose transporters but was not phosphorylated or further metabolized to reflect regional glucose uptake rates and allow to determine alterations in glucose transport [7]. Fluorescently-labeled glucose analogs have been explored to provide biochemical tools for assessing differences in glucose uptake efficiency in vitro [8,9]. Targeting glucose GLUTs has also been broadly explored for disease therapy, with various glycoconjugates synthesized with the goal of improving the specificity of conventional therapeutics [10,11]. While some cases have documented positive trends toward achieving disease-specific activity of therapeutic agents, the majority of efforts were challenged by the global presence of glucose transporters in all tissues. This limitation has promoted the search for new, more disease-specific targets.

Over the last decade, significant dependence on fructose has been established for many cancers [12]. Specific links between high fructose uptake and cancer provided a basis for exploiting GLUT-targeting chemical tools for disease detection and therapy. While several GLUTs have been identified to transport fructose (GLUTs 2, 5, 7, 9, 11, and 12), GLUT5 shows a unique specificity to fructose, with other fructose transporters being capable of transferring glucose and other monosaccharides [13]. In addition to the unique substrate specificity, GLUT5 shows a tightly regulated expression in healthy tissues and is primarily present in the intestine, kidney, and brain microglia [14]. Concerning the disease, GLUT5 was found to gain expression in various cancers that develop from epithelial cells while absent in their healthy counterparts [14]. As a result, radiolabeled fructose analogs have been sought to target fructose-dependent cancer cells. Thus far, the design of the PET imaging probe aimed at targeting fructose transport has been limited to the fluoro derivatives of fructose and 2,5-anhydro-D-mannitol (a furanose analog of fructose) [15,16,17]. Despite the diversity of structures, the direct fructose derivatives were found to be not competitive enough to induce effective accumulation of the radiolabel in cancer cells [18], presumably due to the competition from nutritional fructose or a rapid metabolism-coupled excretion of probes in vivo. The current limitations faced in utilizing GLUT5 for cancer-specific imaging imply the need for more effective analogs that could compete with nutrient sugars for the uptake through the sugar transport targeting.

The growing understanding of the links between fructose uptake and metabolic diseases led several research groups, including ours, to develop molecular probes to target GLUT5 in live cells [18,19,20,21]. It has been demonstrated that GLUT5 has the capacity to bind and transport non-native moieties, such as small fluorophores or bioactive compounds, in the form of glycoconjugates of 2,5-anhydro-D-mannitol (2,5-AM) [19,22,23]. Analysis of diverse coumarin glycoconjugates (ManCous) has revealed an intriguing possibility to tune the strength of probe interaction with GLUT5 through coumarin functionalization while maintaining GLUT5-specificity [19].

**Figure 1:**
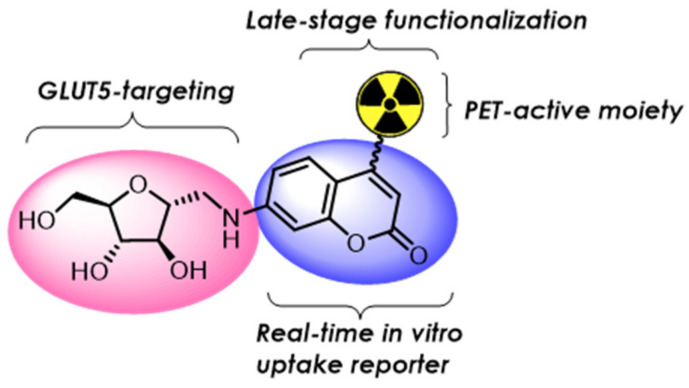
GLUT5-specific probe for delivering the fluoride for PET imaging.

Here, we report a ManCou-based fluoride-functionalized probe that specifically targets GLUT5 with high affinity and exhibits effective GLUT5-dependent uptake in the presence of nutrient fructose (Figure 1). The probe design enables late-stage functionalization and supports the extension of the current synthetic methodology to obtain ^18^F-analog for future GLUT5-specific in vivo cancer imaging applications.

## 2. Results

### 2.1. Design and Synthesis

The chemical modifications of coumarins have been widely explored due to their vast application as dyes and medicinal agents [24]. Numerous synthetic approaches for C-F bond formation have been reported, with some being applicable to the introduction of a radioactive ^18^F [25]. The major challenge in developing PET imaging probes is that modification with fluoride must be done at the last step of the chemical synthesis and should not require excessive purification. This limitation is set by the short half-life of the radioactive ^18^F (the activity decays by half every 109.7 min). While we were developing a synthetic strategy for the fluoride-substituted ManCou probe, ways to introduce fluoride at the last step of the synthesis were explored. We have envisioned that the utilization of the orthogonal reactivity of the azide towards an alkyne will allow us to produce the fluorinated ManCou analog—ManCou-F (Figure 1) [25]. The “click reaction” [26] between these two functional groups is very specific and can be performed in the presence of different functional groups (including hydroxyls of sugars). Moreover, this procedure has also been validated for the introduction of the ^18^F label using fluoro-derivatized alkynes [25]. To introduce the fluoride, we have used 5-fluoro-1-pentyne since it is a synthetically accessible fluorinated alkyne. Within these proof-of-concept studies, we have limited the alkyne selection to the smaller substrate considering our prior observations of stringent requirements in substrate size and hydrophilicity for maintaining GLUT5-mediated uptake [22].

**Scheme 1:**
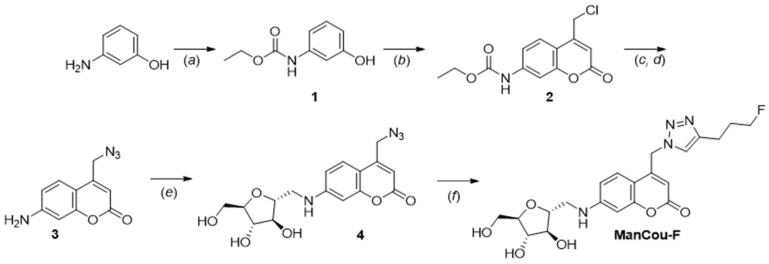
Synthetic route to ManCou-F. Reagents and conditions: (a) ethyl chloroformate (0.5 eq.), reflux, 30 min, 89%; (b) ethyl 4-chloroacetoacetate (1 eq.), H_2_SO_4_, rt, 4 h, 84%; (c) AcOH, H_2_SO_4_, 125 °C, 2 h, 79%; (d) NaN_3_ (4 eq.), dry DMF, Ar, rt, 7 h, 78%; (e) 2,5-anhydro-2-carbaldehyde-D-mannitol (2 eq.), NaBH_3_CN (4 × 0.8 eq.), AcOH, MeOH, rt, 21 h, 90%; (f) 5-fluoro-1-pentyne (5 eq.), CuI (2 eq.), sodium ascorbate (15 eq.), DIEA (15 eq.), CH_3_CN:H_2_O 1:1 (*v*/*v*), rt, 2 h, 30%.

Accessing ManCou-F relies on employing 4-azidomethyl-7-aminocoumarin conjugate of 2,5-AM (**4**) as a starting material for late-stage click chemistry functionalization. The synthesis of the probe was carried out as delineated in Scheme 1. The N-protected 7-amino-4-cloromethylcoumarin **2** was obtained through acid-catalyzed Pechmann condensation [27] between the ethyl chloroformate and N-protected 3-aminophenol **1** that was prepared according to the reported procedure. Azidocoumarin **3** was obtained from the corresponding N-protected 7-amino-4-chloromethylcoumarin **2** through chlorine displacement with nucleophilic azide following the cleavage of the carbamate under acidic conditions [28,29]. Next, the azidocoumarin **3** was transformed into the glycoconjugate **4** through the reductive amination with 2,5-anhydro-2-carbaldehyde-D-mannitol that was obtained according to the reported procedure [19]. ManCou-F was synthesized using 5-fluoro-1-pentyne [30] as a source of fluoride through a copper-catalyzed click reaction [31]. The reaction was completed at room temperature within 2 h, providing the desired triazole conjugate in 30% yield after HPLC purification. Neither the yields nor reaction times were optimized. The identity and purity of all products were confirmed by NMR and ESI-HRMS analysis (Figures S8–S17).

### 2.2. ManCou-F Exhibits Retention in Cells

**Figure 2:**
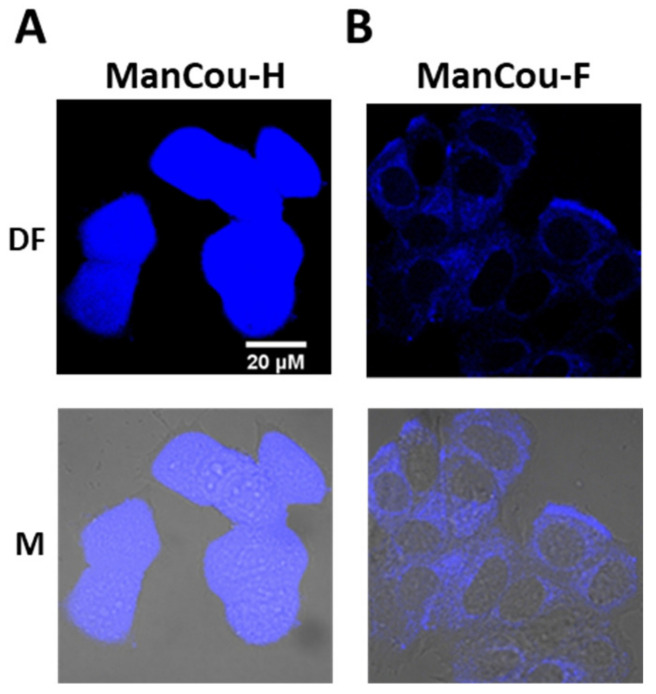
Confocal fluorescence images of MCF7 cells treated with 25 µM of GLUT5 activity reporter ManCou-H (**A**) and ManCou-F (**B**). Fluorescence images were obtained using an Olympus FluoView^TM^ FV1000 confocal microscope and 60× objective. Images were acquired using a DAPI filter (exc/em: 405 nm/461 nm), DF—dark field, and M—fluorescence overlay images with the bright field. The scale bar for all images is 20 µm.

The uptake of the new ManCou-F probe was investigated using a GLUT5-positive breast adenocarcinoma MCF7 cell line (early stage of cancer). Among different cancer types, breast cancer has been found to show dependence on fructose for growth and express GLUT5 at levels altering based on the cancer subtype [32]. The GLUT5 activity in MCF7 cells was validated using ManCou-H [19] as an activity reporter (Figure 2A). ManCou-H-induced fluorescence was observed, reflecting the evident presence of GLUT5 in the MCF7 cells.

To identify whether ManCou-F passes into the cell, MCF7 cells were incubated with the probe for a short period of time (15 min, 37 °C). The short time interval was selected to monitor the transporter-assisted uptake. ManCou-F was used at 25 µM concentration due to the prominent levels of fluorescence observed at this concentration with the prior ManCou analogs. After 15 min of incubation, the probe solution was removed, cells were washed and supplemented with culture media and imaged using a confocal microscope, 60× objective, and DAPI filter. The appearance of blue fluorescence in cells reflected the uptake of ManCou-F in MCF7 cells (Figure 2B).

### 2.3. ManCou-F Passes to the Cells in a Concentration-Dependent Manner

**Figure 3:**
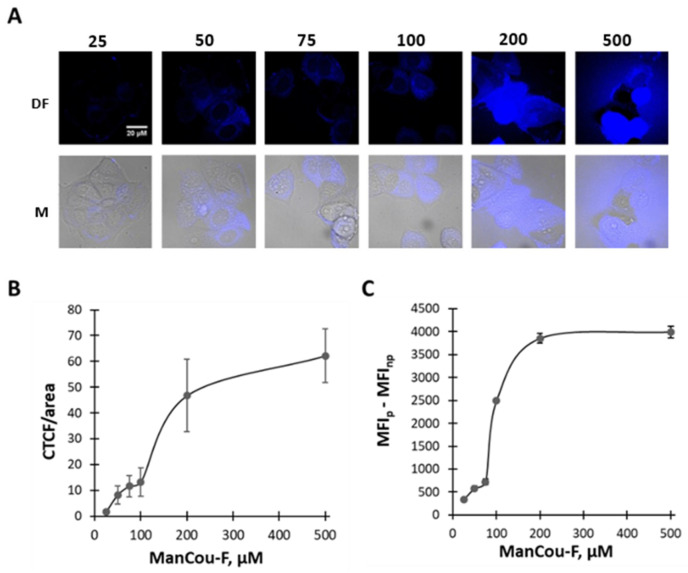
Uptake analysis of different concentrations of ManCou-F (**A**) by confocal microscopy (**B**) and flow cytometry (**C**). (**A**) Representative confocal fluorescent images of concentration-dependent uptake of the probe by MCF7 cells. Images were acquired using a 60× objective and DAPI filter (exc/em: 405 nm/461 nm) at the same laser intensity and exposure time, DF—dark field, and M—fluorescence overlay images with the bright field. Each experiment was carried out in duplicate. (**B**) CTCF quantitated fluorescence. Quantitative fluorescence data were derived from fluorescence images of probe-treated cells using ImageJ as CTCF/area (CTCF = (IntDens (cell)) − (Int Den (bgr)) × Area (cell)). For confocal analysis, data were derived for two images per sample (~5–10 cells/image). Error bars represent the standard deviation of cell fluorescence. (**C**) Fluorescence obtained by Attune NxT Flow Cytometer using VL1 filter was processed as MFI_probe_ (median of fluorescence intensity)-MFI_np_ (MFI of the cells with no treatment). MFI was derived excluding debris and doublets by FSC-SSC and FSC-H-FSC-W gating, respectively. For flow analysis, data were derived for 10,000 events. Each experiment was carried out in duplicate. Error bars represent the standard deviation between two experiments. The scale bar for all images is 20 µm.

We further carried out the analysis of the probe uptake over a range of probe concentrations (to evaluate the uptake kinetics). For the analysis, a 10 mM stock solution in DMSO was used and serial dilutions using a complete culture medium were performed to reach the target concentration (25–500 µM). The overall concentration of DMSO was kept at ≤5%. For the initial uptake analysis, MCF7 cells were treated with increasing concentrations of the probe, and probe-induced fluorescence was imaged using confocal microscopy (Figure 3). We measured the uptake of the probe to exhibit concentration dependence and saturate around 200 μM concentration. Using the Michaelis–Menten kinetic model (GraphPad Prism 9.4.1), the K_m_ ~323 μM and ~231 μM (R_sq_ = 0.86) were derived for the uptake measured via confocal microscopy and flow cytometry, respectively. Considering that the uptake is a multistep process that includes probe binding to the transporter, translocation through the transporter, and release into the cell, we also analyzed the uptake kinetics using the allosteric sigmoidal model. The latter model provided a better fitting (R_sq_ = 0.98) with the K_m_ ~92 μM and 105 μM for flow and confocal settings, respectively. Overall, the K_m_ values reflect ~30–100-fold higher uptake efficiency of ManCou-F probe uptake over fructose (K_m_~11 mM [33]).

We further assessed the ability of the probe to remain in the cell and the overall stability of probe-induced fluorescence. Consequently, probe efflux was evaluated by measuring fluorescence amassed in the dye-free complete culture media over 2 min, 15 min, and 60 min post-incubation with ManCou-F. In parallel, monitoring the changes in probe-induced cell fluorescence after 2 min, 15 min, and 60 min post-incubation with ManCou-F was used to assess probe stability in the cell. As depicted in Figure S1, fluorescence was measurable in a dye-free culture media sample collected from probe-treated cells 2 min post-incubation. The fluorescence level for samples collected 15 min post-incubation did not show any increase in fluorescence, suggesting insignificant excretion of the probe from cells. Moreover, the fluorescence measured for samples acquired 1 h post-incubation reduced as compared to 2 min and 15 min samples, suggesting a further uptake of the probe from the solution. Monitoring fluorescence of probe-treated cells showed no significant difference between 2 min and 15 min. Cell fluorescence, however, dropped significantly for the 1 h sample, suggesting the lack of probe leaching to be driven by the decomposition of the probe (fluorophore) in the cell.

### 2.4. ManCou-F Exhibits Metabolism-Coupled Uptake and GLUT5 Preference

**Figure 4:**
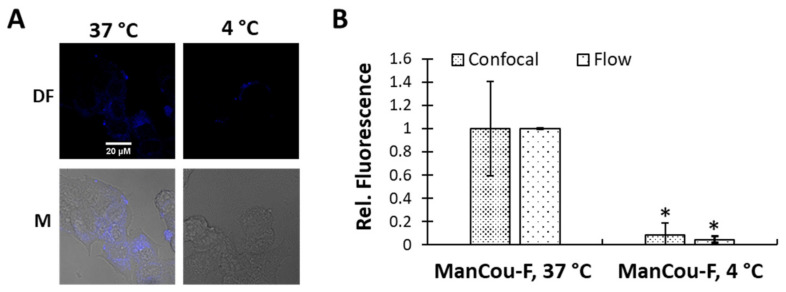
ManCou-F (25 µM) uptake in MCF7 cells preconditioned and treated at 37 °C vs. 4 °C for 15 min. Fluorescence was analyzed by confocal microscopy and flow cytometry. (**A**) Representative images were acquired using 60× objective and DAPI filter (exc/em: 405 nm/461 nm) at the same laser intensity and exposure time, DF—dark field, and M—fluorescence overlay images with the bright field. (**B**) Quantification of fluorescence derived from corresponding fluorescence images using ImageJ as CTCF/Area. A total of two images were analyzed per sample per experiment (~5–10 cells/image). Fluorescence obtained by Attune NxT Flow Cytometer using VL1 filter was processed as MFI_probe_ (median of fluorescence intensity)-MFI_negative_ (MFI of the cells with no treatment). MFI was derived excluding debris and doublets by FSC-SSC and FSC-H-FSC-W gating, respectively. Each experiment was carried out in duplicate. Error bars represent the relative standard deviation derived by dividing the individual standard deviation by the average. A two-tailed *t*-test was used to detect statistically significant differences: * *p* < 0.05. Levels of probe uptake were corrected by the corresponding value obtained for 37 °C to represent relative fluorescence. The scale bar for all images is 20 µm.

We have assessed the involvement of GLUTs in ManCou-F uptake by exploring the connection between cell metabolism and GLUT-mediated uptake. As sustainable GLUT-mediated transport is coupled with sugar phosphorylation [34,35], lowering the metabolic activity of the cell has been shown to also lower the efficiency for GLUT-mediated uptake [19,20,36]. To assess this metabolic connection, MCF7 cells were pre-incubated at 4 °C for 30 min to diminish cell metabolism and further treated at 4 °C with a ManCou-F probe. After treating these cells with a 25 μM concentration of the probe for 15 min and the removal of the probe-containing media, the fluorescence was measured using confocal microscopy (Figure S2A). The cells were then detached from the glass-bottom dish using trypsin and collected, and cell fluorescence was analyzed using flow cytometry (Figure S2B). The comparative analysis of uptake for ManCou-F at 37 °C vs. 4 °C in two analytical settings is summarized in Figure 4. For comparative analysis, the average fluorescence intensity values were corrected by the fluorescence of the control and expressed as relative fluorescence. In both measurement settings, we observed a 90% decrease in the probe uptake for 4 °C samples, indicating the GLUT-mediated and metabolism-coupled uptake for ManCou-F.

**Figure 5:**
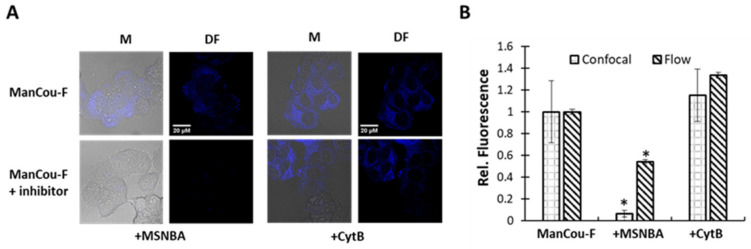
ManCou-F uptake (25 µM) in the presence of MSNBA (25 µM) and cytochalasin B (CytB, 25 μM). (**A**) Images were acquired using a 60× objective and DAPI filter (exc/em: 405 nm/461 nm) at the same laser intensity and exposure time, DF—dark field, and M—fluorescence overlay images with the bright field. Each experiment was carried out in duplicate. (**B**) Analysis of probe-induced fluorescence by confocal microscopy and flow cytometry. For microscopy analysis, data represent fluorescence derived after quantification of fluorescence images using ImageJ as CTCF/Area. Fluorescence obtained by Attune NxT Flow Cytometer using VL1 filter was processed as MFI_probe_ (median of fluorescence intensity)-MFI_negative_ (MFI of the cells with no treatment). Error bars represent the relative standard deviation. A two-tailed *t*-test was used to detect statistically significant differences: *—*p* < 0.05. Relative fluorescence is derived by normalization by the control (probe without inhibitor). The scale bar for all images is 20 µm.

We further moved to establish a specific path for ManCou-F uptake. To assess the involvement of fructose-dependent uptake in the intracellular accumulation of ManCou-F, the probe uptake was evaluated in the presence of increasing concentrations of fructose (25–100 mM, Figure S3). The ManCou-F uptake inhibition was amplified with increasing fructose concertation, reflecting the participation of fructose GLUTs in the uptake of the probe. The inhibitory concentration for fructose against ManCou-F was derived as IC_50_ ~28 mM using non-linear regression analysis. With the implication of fructose GLUTs as a transport path for ManCou-F, we have used specific inhibitors of two major fructose-GLUTs in MCF7 cells to delineate the contribution from each transporter. For GLUT5 inhibition, we used a high-affinity GLUT5-specific inhibitor MSNBA (K_i_ (fructose) ~3.2 μM) [33]. To assess the involvement of non-specific GLUTs, including non-specific fructose transporters GLUT2 and GLUT12 present in MCF7 cells, we used cytochalasin B (CytB) (K_i_ (glucose) ~6.6 μM for GLUT2) [37,38]. For the competitive uptake experiment, MCF7 cells were treated with the combination of a probe with each inhibitor using corresponding complete culture media solutions. ManCou-F was used at 25 μM concentration. All inhibitors were used at concentrations ≥K_i_ to ensure competition with ManCou-F. The probe uptake without the inhibitor was used as a control. The analysis of inhibitor-induced alterations in the probe-induced cell fluorescence was performed using confocal microscopy and flow cytometry (Figure 5). As a result of competition studies, we measured a dramatic drop in ManCou-F uptake in the presence of MSNBA in both confocal and flow settings (Figure S4). In contrast to MSNBA, we observed no inhibition of ManCou-F uptake in the presence of CytB (Figure S5). Overall, the observed inhibition of the uptake by fructose and MSNBA suggested the involvement of fructose transporter GLUT5 in the uptake of ManCou-F. Furthermore, the lack of impact from CytB allowed excluding the participation of non-specific GLUTs, including a second major fructose transporter GLUT2 in the uptake of this probe.

**Figure 6:**
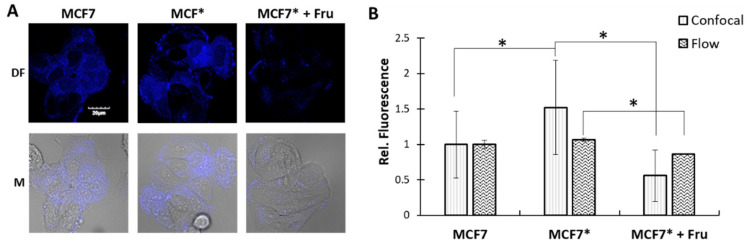
ManCou-F (50 µM) uptake changes with GLUT5 levels and fructose concentration. MCF7: cells cultured in RPMI-1640 with 10% FBS; MCF7*: MCF7 cells cultured with 11 mM fructose for 25 days. MCF7* + Fru: 11 mM fructose supplemented to the MCF7* culture directly with the probe. (**A**) Images were acquired using 60× objective and DAPI filter (exc/em: 405 nm/461 nm) at the same laser intensity and exposure time, DF—dark field, and M—fluorescence overlay images with the bright field. Each experiment was carried out in duplicate. (**B**) Data represent fluorescence derived after quantification of fluorescence images using ImageJ as CTCF/Area. Fluorescence obtained by Attune NxT Flow Cytometer using VL1 filter was processed as MF_Iprobe_ (median of fluorescence intensity)-MFI_negative_ (MFI of the cells with no treatment). MFI was derived excluding debris and doublets by FSC-SSC and FSC-H-FSC-W gating, respectively. Error bars represent the relative standard deviation. A two-tailed *t*-test was used to detect statistically significant differences: * p < 0.05. Relative fluorescence is derived by normalization by the control (MCF7 cells). The scale bar for all images is 20 µm.

To confirm the relationship between GLUT5 activity and ManCou-F uptake, we have assessed ManCou-F uptake in cells with altered levels of GLUT5 expression. For this part, we have relied on the ability of fructose to induce GLUT5 expression [39] and used fructose-preconditioning to increase GLUT5 levels in MCF7 cells [36]. Subsequently, MCF7 cells were grown in the complete RPMI culture media supplemented with 11 mM fructose to establish a fructose-fed MCF7* culture. In parallel, a separate batch from the same starting culture was maintained in the normal complete RPMI culture media to provide the corresponding control (MCF7). MCF7 and MCF7* were propagated in the respective media for 25 days, at which point a ~1.4-fold increase in the GLUT5 expression for MCF7* was measured using immunofluorescence (Figure S6A). To assess the overall differences between the uptake on MCF7 vs. MCF7* cells, the ManCou-F probe was introduced in the normal complete culture media. Probe uptake was measured using confocal microscopy and flow cytometry. Using confocal microscopy, we measured a ~1.5-fold increased probe uptake in fructose-preconditioned MCF7* cells (Figure S6B), confirming the relationship of the probe uptake with the GLUT5-dependent transport. The uptake of ManCou-F in MCF7* cells was, expectedly, inhibited by fructose B (Figure 6B and Figure S7). The overall inhibitory impact from fructose on ManCou-F uptake in MCF7* cells exceeded that observed with MCF7 cells, with 60% inhibition measured in the presence of 11 mM fructose. It is noteworthy that we were unable to detect the increase in GLUT5 activity for MCF7* cells using flow cytometry (Figure 6). However, we observed a statistically significant inhibition of ManCou-F uptake in MCF7* cells in the presence of fructose.

### 2.5. Cytotoxicity Analysis

**Figure 7:**
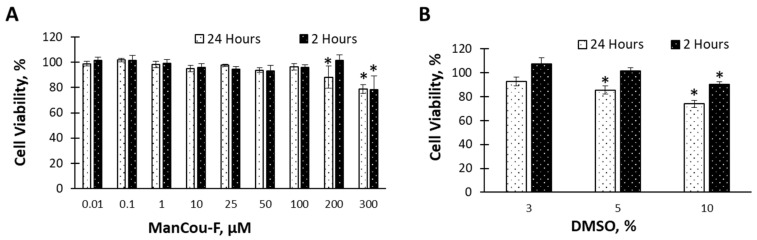
Cell viability of MCF7 cells was assessed using an MTS assay in 96-well plate format and reported as a relative colorimetric change measured at 490 nm. (**A**) ManCou-F cytotoxicity in MCF7 after 2 and 24 h of incubation. Each experiment was carried out in duplicate. (**B**) DMSO cytotoxicity in MCF7 after 2 and 24 h of incubation. A two-tailed *t*-test was used to detect statistically significant differences: * p < 0.05. Error bars represent the standard deviation.

Application of potential PET-imaging agents requires the probe to be non-cytotoxic for the target cells. Hence, we have evaluated the cytotoxicity of ManCou-F using an MTS assay. The cytotoxic responses were assessed for MCF7 cells using increasing concentrations of the probe and continuous 24 h incubation. For analysis, a 10 mM stock solution in DMSO was used, and serial dilutions using a complete culture medium were performed to reach the target concentration (0.01–300 μM). The overall concentration of DMSO was kept at ≤3%. As depicted in Figure 7, at the prolonged 24 h incubation time, ~15% loss in cell viability could be observed at concentrations ≤200 μM. The shorter treatment intervals (2 h) showed no alterations in the cell viability of probe-treated cells up to 300 μM concentration (Figure 7). Considering that increasing ManCou-F concertation is accompanied by an increase in DMSO concertation, we also evaluated the potential impact that DMSO may have on MCF7 cells. For this part, MCF7 cells were treated with complete culture media containing 3, 5, and 10% DMSO, which corresponds to ManCou-F sample concentrations of 300 μM, 500 μM, and 1000 μM, respectively. As depicted in Figure 7B, we measured ~10–15% loss in cell viability for 3% and 5% DMSO samples and >20% loss in viability for the 10% DMSO sample. Overall, comparing the cytotoxicity of ManCou-F with that of DMSO, it appears that the probe is not cytotoxic for MCF7 cells and the observed loss in cell viability for high concentrations of ManCou-F is primarily contributed by DMSO.

## 3. Discussion

Facilitative GLUT transporters have been viewed as important therapeutic targets for several decades. Despite years of research focusing on utilizing GLUTs for disease diagnosis and treatment, the major advances in the field are limited to the development and application of **^18^**F-labeled glucose analog for PET imaging. However, GLUTs targeting still lacks specificity, safety, and efficiency. For example, the ^18^F-labeled analog of 2-fluoro-2-deoxy-D-glucose (2-^18^F-FDG) probe is used as a radiotracer for PET to assess disease-linked alterations in glucose metabolism. Nevertheless, false positive results are common, as increased glucose metabolism is also observed if infectious/inflammatory conditions are present, leading to 2-^18^F-FDG being internalized through the ubiquitously present in all tissues GLUTs to these regions as well [40]. Therefore, more specific targeting is desired to be diagnostically beneficial.

Multiple attempts to use the differences in GLUT activity between normal and metabolically compromised cells have helped to build an understanding of the critical importance of targeting disease-relevant GLUTs in achieving cell discrimination. In this view, fructose-specific transporter GLUT5 has been set as an attractive target since the levels of this transporter were found to alter between normal and cancer cells. Moreover, alterations in GLUT5 activity were observed during cancer development, progression, and metastasis [2]. Particularly, differences in GLUT5 activity were observed for breast cancer cells providing the basis for using GLUT5-targeting to identify cancer cells [41,42]. As a result, GLUT5 targeting has been assessed for developing imaging tools.

To date, the development of radiolabeled fructose analogs to target fructose transport in cells is limited to the direct derivatization of fructose and 2,5-anhydro-D-mannitol (2,5-AM, a furanose analog of fructose) with fluoride [15,16,18,43]. Currently, several fructose analogs have been synthesized and evaluated as potential PET imaging agents. 6-[^18^F]-fluoro-6-deoxy-D-fructose (6-FDF) [16] was shown to efficiently pass into the murine EMT-6 and human breast cancer MCF7 cells, although its rapid metabolism led to a fast decrease in its cellular concentration. 1-[^18^F]-Fluoro-1-deoxy-D-fructose (1-^18^FDF) [17] was tested as a fructose uptake tracer in mouse and rat tumor xenografts and showed rapid clearance through the kidney and liver. 1-[^18^F-fluoro]-1-deoxy-2,5-anhydro-D-mannitol (1-^18^FDAM) was found to accumulate in breast tumors in rabbits but showed rapid excretion [43]. 3-(^18^F)fluoro-3-deoxy-D-fructose (3-^18^FDF) demonstrated fructose-dependent uptake, warranting further evaluation in animals [15]. While all these fructose analogs exerted fructose-dependent uptake, their potencies differed, with 6-FDF showing higher accumulation levels over 1-FDF or 1-FDAM. The observed differences in cellular uptake levels point out the importance of stereochemistry and substitution patterns for the recognition and transport of radiotracers 1-[^18^F]FDAM, 1-[^18^F]FDF, 6-[^18^F]FDF by GLUT5. The relatively similar binding affinities to fructose suggested the possibility of limited competition between fructose and the radiotracer in vivo, reflecting the need for more potential GLUT5 binders for PET agent development.

In our previous studies, we have established the feasibility of enhancing the efficacy of GLUT5 interaction with the substrate through the stabilization of H-bonding and Van der Waals interactions [19]. Namely, derivatization of 2,5-AM with coumarins resulted in GLUT5-specific glycoconjugates that showed enhanced uptake efficacy [44] and competed with fructose at a 1:1000 probe-to-fructose ratio. With the drastic improvement in the competitiveness for GLUT5, we envisioned the ManCou scaffold to support the development of higher affinity PET imaging agents. With the late-stage fluorination in mind, the probe design relied on introducing the fluoride-containing moiety at the last step of chemical synthesis through the click reaction. The final fluorination step between ManCou-N_3_ and 5-fluoro-1-pentyne proceeded in 30% yield.

The resulting fluoride-carrying ManCou-F probe was found to have K_m_ values > 30-fold higher than those of fructose (K_m_ = 11 mM [33]), which allowed for the effective uptake of the probe in the complete culture media and elevated fructose concentrations. The dependence of probe uptake on cell incubation temperature supported the consideration of GLUT-mediated uptake as a primary path for probe accumulation in cells. The following alteration in probe uptake upon changes in fructose levels in the incubation media and GLUT5 levels in MCF7 cells delineated the uptake to be dependent on fructose GLUTs. The strong impact of GLUT5-specific inhibitor MSNBA highlighted the involvement of GLUT5 in the uptake of ManCou-F. Lastly, the lack of inhibition from cytochalasin B excluded non-specific glucose and fructose GLUTs. In combination, the studies validated the GLUT5-specificity of the probe and the feasibility of achieving the GLUT5-specific delivery of the fluoride into the cell in the presence of the nutrient fructose.

The introduction of a radiolabel at the last stage of the synthesis of ManCou-F provides a possibility to use the described approach in the synthesis of a radiolabeled (^18^F)-ManCou-F analog. The 5-fluoro-1-pentyne utilized in the synthesis of ManCou-F was previously reported to be accessible in the ^18^F-labeled form. The synthetic approaches were reported to rely on the displacement of the OTs-group of 5-(p-toluenesulfonyl)-1-yne using DAST in dichloromethane over 50 min in ~50% radiochemical yield [45]). The synthesis of 5-(^18^F)fluoro-1-pentyne was also reported using K222 in acetonitrile with ~86% radiochemical yield [30]). Using the DAST-mediated fluorination, the 5-fluoro-1-pentyne is distilled out as a solution that is directly used in the subsequent “click” reaction. It is feasible that upon the immediate use of 5-(^18^F)fluoro-1-pentyne with ManCou-N_3_ (**4**), the overall radioactive yield of (^18^F)-ManCou-F could reach ~16% or ~25%, respectively, considering the reported 50% and 86% radioactive yields of 5-(^18^F)fluoro-1-pentyne. The estimated radioactive yield accounts for 120 min “click” reaction time and 30 min purification time using flash chromatography and solvent evaporation. Further optimization of the click reaction time could contribute to improving the radioactive yield of the conjugate.

## 4. Materials and Methods

### 4.1. Cell Culture and Plate Preparation

For all fluorescence analyses, the adenocarcinoma MCF7 cell line (ATCC) was cultured using RPMI-1640. Complete RPMI-1640 medium contained 10% fetal bovine serum (FBS) and 1% penicillin/streptomycin. Fructose-reconditioned MCF7* cells were obtained by propagating MCF7 cells in RPMI, supplemented with dialyzed FBS (10%), 1% penicillin/streptomycin, and 11 mM fructose for 25 days. Cells were cultivated in 10 cm cell culture dishes at 37 °C in 5% CO_2_ and under 65% relative humidity. MCF7 cells were collected at ~80% confluence of the 10 mm tissue culture plate using 0.25% Trypsin-EDTA (2 mL). The trypsin fraction was diluted with culture media to 5 mL; cells were pelleted by centrifuging (1600 rpm, 5 min), reconstituted in the complete culture media (5 mL), and plated with a seeding density of 150,000 cells per 35 mm glass-bottom confocal dishes (MatTek). The total volume in the plate was brought to 2 mL by corresponding media when needed. After 12 h, the culture media was changed, and cells were allowed to grow for 48 h from the moment of cultivation.

### 4.2. General Protocol for Cell Imaging and Fluorescence Image Processing Using a Confocal Microscope

Cell images were taken with Olympus FluoView^TM^ FV1000 using the FluoView software. The 60× oil-suspended lens was used to observe fluorescent activity with the following conditions: DAPI; laser 405 nm (35% intensity); 20 μs/pixel. Images were taken at the same laser intensity and exposure time. The obtained fluorescence images were quantified using ImageJ. Fluorescence was calculated as Corrected Total Cell Fluorescence (CTCF) (CTCF = Integrated Density − (Area of the selected cell × Mean fluorescence of background readings). Next, CTCF was normalized with consideration of the cell area—CTCF divided by the area of the selected cell (CTCF/area of the selected cell). This procedure was performed for cells by selecting regions of interest in the single image. The finalized values represent the average fluorescence of 5–10 cells per image. Per every experiment, a minimum of 2 images were analyzed.

### 4.3. General Protocol for Fluorescence Analysis Using Flow Cytometry

After the confocal microscopy imaging, the cells were then detached from the glass-bottom dish using trypsin (0.5 mL). Next, the trypsin fraction was diluted with culture media to 1 mL and transferred into the microcentrifuge tube (1.5 mL) for cell fluorescence analysis by flow cytometry. Fluorescence data of 10,000 events was obtained using the VL1 filter of the Attune NxT Flow Cytometer. The obtained data were processed as MFI_probe_ (median of fluorescence intensity)-MFI_negative_ (MFI of the cells with no treatment). MFI was derived excluding debris and doublets by FSC-SSC and FSC-H-FSC-W gating, respectively.

### 4.4. Uptake Analysis

For all biological studies, ManCou-F of analytical purity was obtained by reverse-phase HPLC purification. 500 μM solution was prepared from 10 mM DMSO stock solution using the complete cell media for the dilution. Probe solutions (25, 50, 75, 100, and 200 μM) were prepared from 500 μM solution. DMSO concentrations ranged from 0.25 to 5%. For treatment, cells seeded in glass-bottom confocal dishes were used. Cell culture media was removed, and a probe-containing culture media solution (2 mL) was added. Cells were incubated with probes at 37 °C for 15 min. After incubation, the probe solution was removed, and cells were washed with the complete culture media (2 × 1 mL) and replenished with 2 mL of media for fluorescence analysis using a confocal microscope (4.2) and a flow cytometer (4.3).

### 4.5. Temperature Studies

To explore GLUT transporters’ involvement in the probes’ uptake and exclude passive diffusion throughout the membrane, the uptake of probes was evaluated at 4 °C. For this, MCF7 cells were seeded in confocal plates as described in Section 4.1. Before treatment with probes, confocal plates with the seeded cells were kept in a fridge at ~4 °C for 30 min. After that, the culture media were discarded and a cooled solution of a probe in the complete culture media was added. Cells were kept in the fridge at ~4 °C for another 15 min. After treatment, the probes solution was removed, and cells were washed with culture media (2 × 1 mL) and replenished with 2 mL of media for imaging according to Section 4.2 and Section 4.3.

### 4.6. Competitive Uptake Inhibition Studies

For uptake inhibition analysis, the following stock solutions were used: fructose (200 mM in the complete culture media); MSNBA (5 mM in DMSO), and CytB (500 µM in DMSO). For treatment, stock solutions of inhibitors were diluted with the complete culture media to reach the 2× of required concentration and further diluted by the probe-containing solution to reach the targeted inhibitor concentration. For treatment, cells seeded in glass-bottom confocal dishes were used. Cell culture media were removed, and a probe + inhibitor containing culture media solution (2 mL) was added. Cells were incubated at 37 °C for 15 min. After incubation, the probe + inhibitor solution was removed, and cells were washed with the complete culture media (2 × 1 mL) and replenished with 2 mL of media for fluorescence imaging according to Section 4.2 and Section 4.3. Cells treated with 25 µM ManCou-F were used as a control.

### 4.7. GLUT5 Immunofluorescence Analysis

Cells were grown in their respective media and harvested using 0.25% trypsin. After centrifugation, trypsin was discarded, and cells were reconstituted in the complete growth medium, seeded in confocal dishes, and allowed to adhere for 12 h. Then, the cells were fixed with 4% PFA for 20 min, followed by washes with PBS (3 × 2 mL) for a total of 15 min. The cells were then protein blocked using bovine serum albumin, followed by overnight incubation at 4 °C with primary antibody (Santa Cruz Biotechnology, sc-27105) at a dilution of 1:200. Next, the cells were washed with PBS (2 × 2 mL) and incubated for 2 h with the secondary antibody conjugated to Alexa 488 (Invitrogen, Thermo Fisher Scientific, A32723) at a dilution of 1:1000. After the reagents were washed off, cells were imaged using a confocal microscope, using 60× objective and Alexa488 filter. Imaging and analysis were carried out according to the general procedure described in Section 4.2.

### 4.8. Cell Viability Analysis (MTS Assay)

The MCF7 cell pellet, obtained according to Section 4.3, was reconstituted in the complete RPMI medium, seeded in 96-well flat-bottom opaque walled plates (10,000 cells/well), and allowed to grow for 24 h at 37 °C and 5% CO_2_. At the end of 24 h, the media were discarded, and the cells were replenished with media supplemented with ManCou-F (concentrations varying from 0–300 µM) or DMSO (3-10%) and incubated at 37 °C and 5% CO_2_ for 2 and 24 h. Then, 20 µL of CellTiter96 Aqueous one solution cell proliferation assay kit (Promega-G3582) reagent was added directly to each well, and the cells incubated in the incubator conditions for 4 h. Wells with media only were used as the negative control. Untreated cells represent 100% of cell viability. At the end of 4 h, the absorbance was immediately collected using an automated UV 96-well plate reader at 490 nm wavelength. Cell viability of treated cells was calculated as a relative decrease in the absorbance with respect to the untreated control: Viability, % = (A_Treatment_ − A_Control_) × 100 (where A = absorbance). The results represent the mean ± SD of triplicate samples.

### 4.9. Quantification, Statistical, and Kinetic Analysis

Quantified fluorescence is reported as CTCF/cell area. Data are presented as ± standard deviation of the average fluorescence (calculated by Excel) between cells in two independent experiments. Relative fluorescence is derived by dividing the average fluorescence (control or sample) by that of the control. Relative standard deviation is derived by dividing the individual standard deviation by the average standard deviation [46]. Statistical significance was calculated using a 2-tailed *t*-test and is indicated in figures using the following denotation: * *p* < 0.05. Probe uptake kinetic analysis was carried out using enzyme kinetic, velocity as a function of substrate models using GraphPad Prism 9.4.1.

## 5. Conclusions

Overall, we show that GLUT5-specific targeting and delivery of a radiolabeled fluoride is feasible by employing the ManCou scaffold. The application of the click reaction for the late-stage functionalization also allows assessing the ^18^F-labeled analog due to the availability of synthetic approaches to access the [^18^F]-5-fluoro-1-pentyne. While longer fluorinated alkynes are available, we restricted the studies to this shortest representative as GLUT5-mediated uptake was found to be sensitive to the overall size and hydrophobicity of the substrate. The selectivity and high efficacy of uptake measured for ManCou-F warrant further analysis of ManCou-F and the corresponding ^18^F analog in vivo. The outcomes of the studies will be reported in due course.

## Data Availability

Not applicable.

## Supplementary Materials

The following supporting information can be downloaded at: https://www.mdpi.com/article/10.3390/ijms24010173/s1.
