# Supplementary material for: Late-Stage Functionalization through Click Chemistry Provides GLUT5-Targeting Glycoconjugate as a Potential PET Imaging Probe
Source: Int J Mol Sci. 2022 Dec 22;24(1):173. doi: 10.3390/ijms24010173 (PMC9820411; doi:10.3390/ijms24010173)
Supplement: Supplementary file 1 [file ijms-24-00173-s001.zip › ijms-2055629-supplementary.pdf]

Supporting Information

Late-Stage Functionalization through Click Chemistry Provides GLUT5-Targeting Glycoconjugate as a Potential PET Imaging Probe

Adelina Oronova[1,2] and Marina Tanasova[1,2,*]

[1]Department of Chemistry, Michigan Technological University, 1400 Townsend Drive, Houghton, Michigan 49931, USA
[2]Health Research Institute, Michigan Technological University, 1400 Townsend Drive, Houghton, Michigan 49931, USA

*E-mail: mtanasov@mtu.edu

Table of Contents

1. Supplementary Figures 2
2. General Information: Supplies, Methods, and Instrumentation 6
3. Organic Synthesis 7
4. HRMS, ^{1}H and ^{13}C NMR spectra 9
5. References 19

1. Supplementary Figures

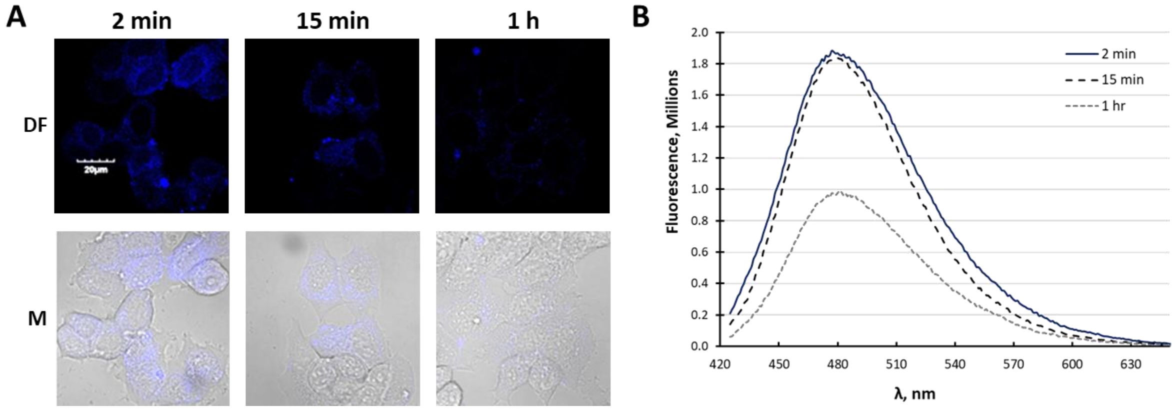

Figure S1: Cellular stability and efflux of ManCou-F. (A) Probe stability was analyzed by imaging fluorescence for ManCou-F-treated cells 2-, 15-, and 60-min post incubation. Fluorescence images of MCF7 cells treated with ManCou-F (Control) and ManCou-F+Fructose. Images were acquired using confocal microscope, 60× objective, and DAPI filter (exc/em: 405 nm/461nm) at the same laser intensity and exposure time, DF – dark field, and M – fluorescence overlay images with the bright field. Scale bar for all images is 20 µm. (B) Fluorescence in the dye-free complete culture media collected from probe-treated cells 2-, 15-, and 60-min post-incubation with ManCou-F. The fluorescence spectra (λ_{ex} = 405 nm) were measured using a Fluoromax-4 spectrophotometer.

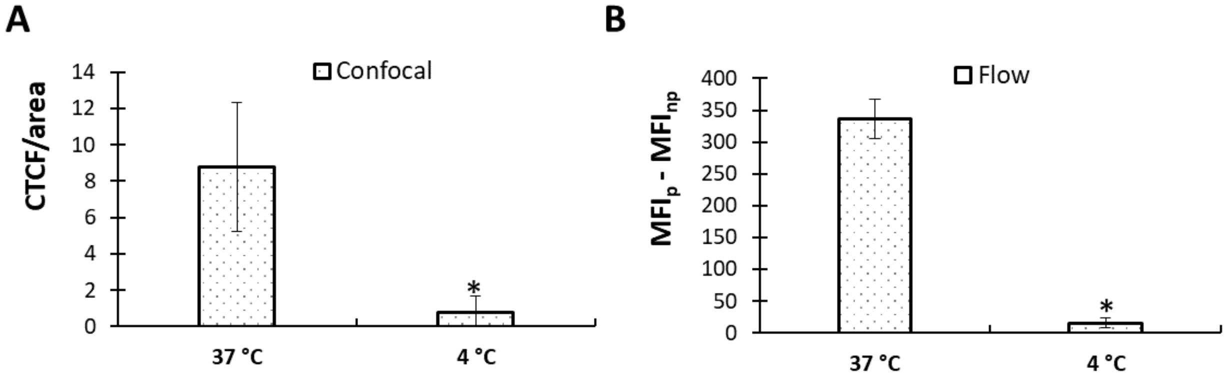

Figure S2: ManCou-F (25 µM) uptake in MCF7 cells preconditioned and treated at 37 °C vs. 4 °C for 15 min. Fluorescence was analyzed by confocal microscopy and flow cytometry. (A) Quantification of fluorescence derived from corresponding fluorescence images using ImageJ as CTCF/Area. A total of 2 images were analyzed per sample (~5-10 cells/image). (B) Fluorescence obtained by Attune NxT Flow Cytometer using VL1 filter was processed as MFI_{probe} (median of fluorescence intensity)-$MFI_{noprobe}$ (MFI of the cells with no treatment). MFI was derived excluding debris and doublets by FSC-SSC and FSC-H-FSC-W gating, respectively. For flow analysis, data were derived for 10,000 events. Error bars represent the standard deviation. A two-tailed *t*-test was used to detect statistically significant differences: *$p < 0.05$. Each experiment was carried out in duplicate.

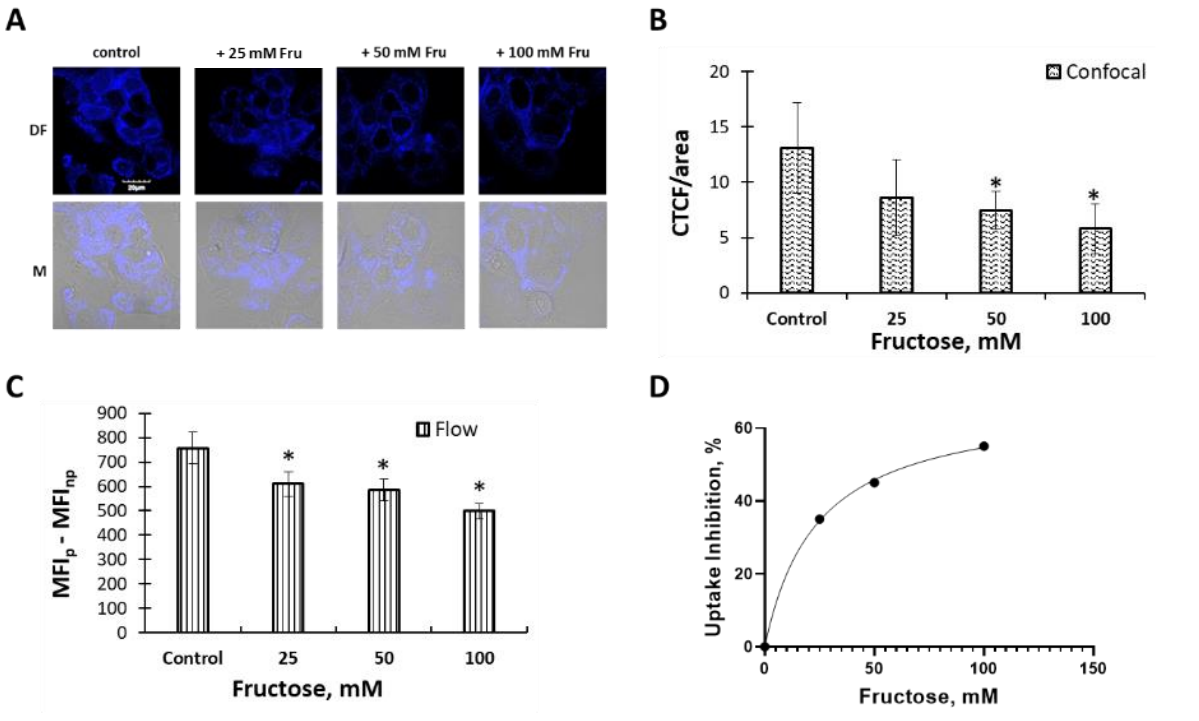

Figure S3: ManCou-F uptake (25 µM) in the presence of 25-100 mM fructose. (A) Fluorescence images of MCF7 cells treated with ManCou-F (Control) and ManCou-F+Fructose. Images were acquired using confocal microscope, 60× objective, and DAPI filter (exc/em: 405 nm/461nm) at the same laser intensity and exposure time, DF – dark field, and M – fluorescence overlay images with the bright field. Scale bar for all images is 20 µm. (B) Quantification of fluorescence derived as CTCF/Area from the corresponding fluorescence images (A) using ImageJ. A total of 2 images were analyzed per sample (~5-10 cells/image). (C) Fluorescence obtained by Attune NxT Flow Cytometer using VL1 filter was processed as MFI_{probe} (median of fluorescence intensity)-$MFI_{noprobe}$ (MFI of the cells with no treatment). MFI was derived excluding debris and doublets by FSC-SSC and FSC-H-FSC-W gating, respectively. For flow analysis, data were derived for 10,000 events. Error bars represent the standard deviation. A two-tailed *t*-test was used to detect statistically significant differences: $*p < 0.05$. Each experiment was carried out in duplicate. (D) ManCou-F uptake inhibition by fructose (GraphPad Prism 9.4.1).

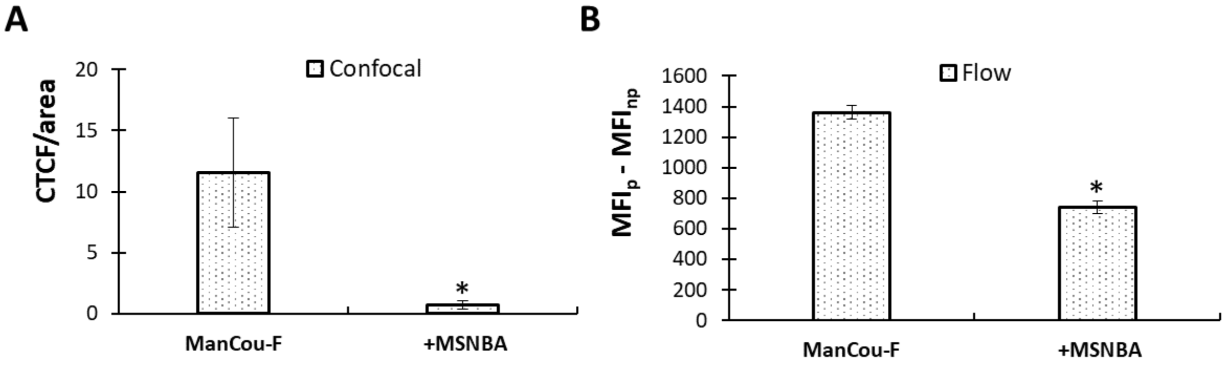

Figure S4: ManCou-F uptake (25 µM) in the presence of MSNBA (25 µM). (A) Quantification of fluorescence derived from corresponding fluorescence images using ImageJ as CTCF/Area. A total of 2 images were analyzed per sample (~5-10 cells/image). (B) Fluorescence obtained by Attune NxT Flow Cytometer using VL1 filter was processed as MFI_{probe} (median of fluorescence intensity)-$MFI_{noprobe}$ (MFI of the cells with no treatment). MFI was derived excluding debris and doublets by FSC-SSC and FSC-H-FSC-W gating, respectively. For flow analysis, data were derived for 10,000 events. Error bars represent the standard deviation. A two-tailed *t*-test was used to detect statistically significant differences: *$p < 0.05$. Each experiment was carried out in duplicate.

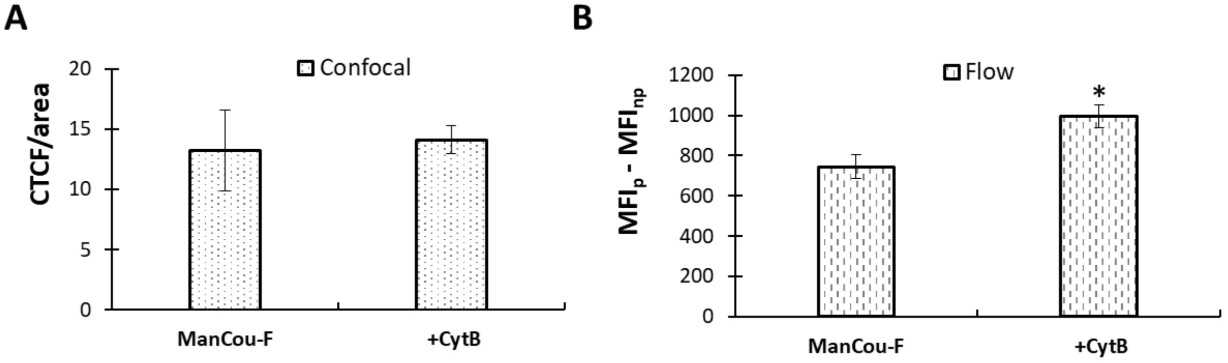

Figure S5: ManCou-F uptake (25 µM) in the presence of cytochalasin B (CytB, 25 µM). (A) Quantification of fluorescence derived from corresponding fluorescence images using ImageJ as CTCF/Area. A total of 2 images were analyzed per sample (~5-10 cells/image). (B) Fluorescence obtained by Attune NxT Flow Cytometer using VL1 filter was processed as MFI_{probe} (median of fluorescence intensity)-$MFI_{noprobe}$ (MFI of the cells with no treatment). MFI was derived excluding debris and doublets by FSC-SSC and FSC-H-FSC-W gating, respectively. For flow analysis, data were derived for 10,000 events. Error bars represent the standard deviation. A two-tailed *t*-test was used to detect statistically significant differences: *$p < 0.05$. Each experiment was carried out in duplicate.

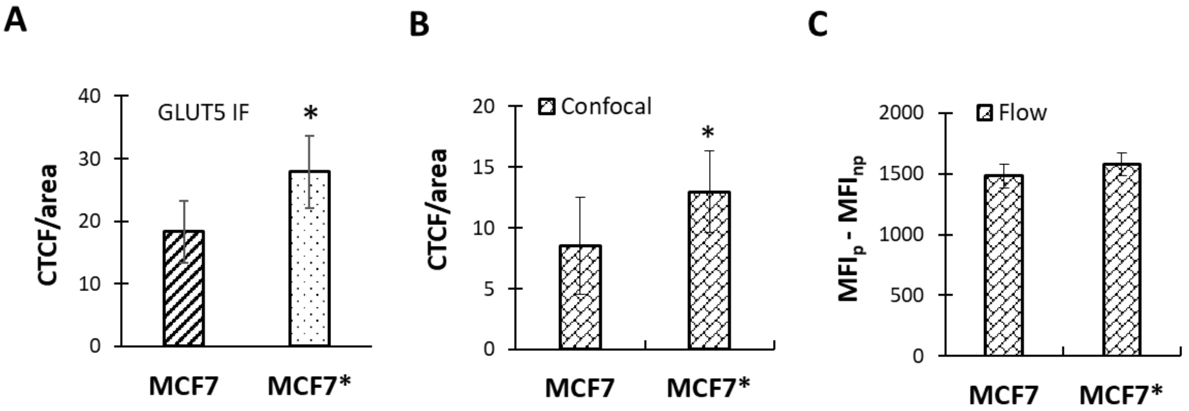

Figure S6: ManCou-F (50 µM) uptake changes with GLUT5 levels and fructose concentration. MCF7: cells cultured in RPMI-1640 with 10% FBS; MCF7*: MCF7 cells cultured with 11 mM fructose for 25 days. (A) Immunofluorescence analysis of GLUT5 expression in MCF7 vs. MCF7*. (B) Quantification of fluorescence derived from corresponding fluorescence images using ImageJ as CTCF/Area. A total of 2 images were analyzed per sample (~5-10 cells/image). (C) Fluorescence obtained by Attune NxT Flow Cytometer using VL1 filter was processed as MFI_{probe} (median of fluorescence intensity)-$MFI_{noprobe}$ (MFI of the cells with no treatment). MFI was derived excluding debris and doublets by FSC-SSC and FSC-H-FSC-W gating, respectively. For flow analysis, data were derived for 10,000 events. Error bars represent the standard deviation. A two-tailed *t*-test was used to detect statistically significant differences: *$p < 0.05$. Each experiment was carried out in duplicate.

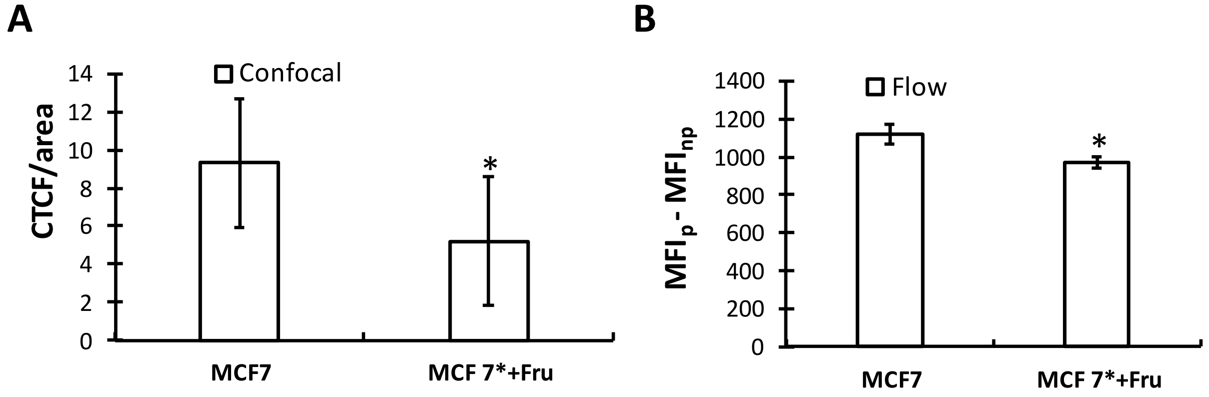

Figure S7: Competition between ManCou-F (50 µM) and fructose identifies involvement of fructose transporters. MCF7: cells cultured in RPMI-1640 with 10% FBS; MCF7*: MCF7 cells cultured with 11 mM fructose for 25 days. MCF7* + Fru: 11 mM fructose supplemented to the MCF7* culture directly with the probe. (A) Quantification of fluorescence derived from corresponding fluorescence images using ImageJ as CTCF/Area. A total of 2 images were analyzed per sample (~5-10 cells/image). (B) Fluorescence obtained by Attune NxT Flow Cytometer using VL1 filter was processed as MFI_{probe} (median of fluorescence intensity)-$MFI_{noprobe}$ (MFI of the cells with no treatment). MFI was derived excluding debris and doublets by FSC-SSC and FSC-H-FSC-W gating, respectively. For flow analysis, data were derived for 10,000 events. Error bars represent the standard deviation. A two-tailed *t*-test was used to detect statistically significant differences: $*p < 0.05$. Each experiment was carried out in duplicate.

2. General Information: Supplies, Methods, and Instrumentation

Chemical synthesis supplies and analytical methods: All reagents were used as received unless otherwise stated from Sigma-Aldrich (St Lois, MO, USA), TCI America (New Jersey, NJ, USA), Alfa Aesar (Haverhill, MA, USA), Ark Pharm (Arlington Heights, IL, USA), or Chem-Impex International (Wood Dale IL, USA). Analytical TLC was carried out on commercial SiliCycle SiliaPlate® 0.2 mm F254plates. Preparative silica chromatography was performed using SiliCycle SiliaFlash® F6040–63 m (230–400 mesh). The final purification of compounds was achieved with Agilent-1200 HPLC (high-pressure liquid chromatography) using a reverse-phase semi-preparative column (Phenomenex® Luna® 10 mC18(2) 100 Å, LC Column 100, 10 mm, Ea). ^{1}H, ^{13}C, and ^{19}F NMR spectra were recorded at room temperature with a Varian Unity Inova 400 MHz spectrometer. CD_3OD, and DMSO-d_6 were used as solvents and referenced to the corresponding residual solvent peaks (respectively; 3.31 and 49.0 ppm for CD_3OD, respectively; 2.50 and 39.52 ppm for DMSO-d_6, respectively) [31]. The following abbreviations are used to indicate the multiplicity: s—singlet; d—doublet; t—triplet; q—quartet; m—multiplet; b—broad signal; app—approximate. The coupling constants are expressed in Hertz (Hz). The high-resolution (HR) MS data (ESI) were obtained using a Thermo Fisher Orbitrap Elite™ Hybrid Ion Trap-Orbitrap Mass Spectrometer at Chemical Advanced Resolution Methods (ChARM) Laboratory at Michigan

Technological University. Fluorescence spectra were obtained with FluoroMax-4 spectrophotometer.

Cell culture supplies and analytical methods: MCF7 cells were purchased from ATCC (Manassas, VA, USA) and cultures according to the recommended protocol. RPMI-1640, dye-free RPMI, Penicillin/Streptomycin, FBS (Fetal Bovine Serum), dialyzed FBS, and 0.25% Trypsin-EDTA (1X) were purchased from Life Technologies, USA. 10 cm cell culture dishes treated for increased cell attachment and poly-D-Lysine coated 35 mm confocal plates were purchased from VWR and MatTek, respectively. Sterile DMSO (25-950-CQC, 250 mL) was purchased from Sigma-Aldrich (St Lois, MO, USA). CellTiter 96® Aqueous One Solution Cell Proliferation Assay was purchased from Promega (Madison, WI, USA). Confocal images were taken with Olympus FluoView™ FV1000 using the FluoView software. Flow cytometry analysis was performed using Attune NxT flow cytometer.

3. Organic Synthesis

7-Amino-4-(chloromethyl)-2*H*-chromen-2-one[1], 2,5-anhydro-2-carbaldehyde-D-mannitol[2], and 5-fluoro-1-pentyne (as ca. 10% solution in CH_2Cl_2)[3] were prepared according to the reported procedures. Other compounds were synthesized as outlined below.

7-Amino-4-(azidomethyl)-2H-chromen-2-one (**3**). This compound was prepared based on the reported procedure[4]. To the solution of 7-Amino-4-(chloromethyl)-2*H*-chromen-2-one[1] (2.3 g, 11.0 mmol, 1 eq.) in dry DMF (30 mL) under Ar, NaN_3 (2.85 g, 43.8 mmol, 4 eq.) was added, and the mixture was stirred at room temperature for 4 h. The mixture was diluted with EtOAc (400 mL), washed with brine (4 x 100 mL), and dried with $MgSO_4$. After filtration and concentration, the residue was absorbed on silica gel and purified by column chromatography on silica gel, eluting with 0−10% methanol in CH_2Cl_2. The resulting solid was suspended in CH_2Cl_2, followed by the addition of hexanes and filtered, washing with hexanes, to provide **3** as a dark yellow solid in 78% yield (1.86 g). Prolonging the reaction time was found to be detrimental to yields. HRMS (ESI): m/z $[M + H]^+$ calc'd for $C_{10}H_9N_4O_2$: 217.07257; found: 217.07222. ^{1}H NMR (400 MHz, DMSO-d_6): δ, 7.37-7.35 (d, J = 8.4, 1H), 6.58-6.55 (dd, J_1 = 2.0, J_2 = 8.8, 1H), 6.43 (d, J = 2.0, 1H), 6.23 (bs, 2H), 6.05 (s, 1H), 4.69 (s, 2H) ppm. ^{13}C NMR (100 MHz, DMSO-d_6): δ, 160.4, 155.6, 153.2, 150.2, 125.6, 111.2, 106.3, 106.2, 98.6, 49.6 ppm.

4-(Azidomethyl)-7-((((2R,3S,4S,5R)-3,4-dihydroxy-5-(hydroxymethyl)tetrahydrofuran-2-yl)methyl)-amino)-2H-chromen-2-one acetic acid salt (**4**). This compound was prepared based on our reported procedure[2]. Crude 2,5-anhydro-2-carbaldehyde-D-mannitol (440 mg, up to 2.7 mmol) and coumarin **3** (223 mg, 1.05 mmol) were dissolved in methanol (35 mL). AcOH (3.5 mL) was added to adjust the pH to <6, and the mixture was stirred at room temperature for 10-15 minutes, followed by portion-wise addition of $NaBH_3CN$ (4 x 52 mg, 0.83 mmol, 0.8 eq. every 30-40 minutes). The reaction mixture was stirred at room temperature for an overall 21 h. The mixture was concentrated to dryness under reduced pressure, absorbed on silica gel, and purified by column chromatography on silica gel eluting with 0−10% methanol in CH_2Cl_2. The product was obtained as an acetic acid salt, yellow foam in up to 90% yield (up to 400 mg), which was used in the

subsequent step without further purification. Exclusively for analytical purposes, the final purification was achieved by semi-preparative HPLC using a water-acetonitrile gradient starting with 20% acetonitrile to obtain the free amine as a yellow foam (40 mg sample). HRMS (ESI): m/z $[M + H]^+$ calc'd for $C_{16}H_{19}N_4O_6$: 363.13050; found: 363.13033. ^{1}H NMR (400 MHz, CD_3OD): δ, 7.38-7.36 (d, J = 9.2, 1H), 6.68-6.65 (dd, J_1 = 2.4, J_2 = 8.8, 1H), 6.50 (d, J = 2.4, 1H), 6.10 (s, 1H), 4.59 (d, J = 1.2, 2H), 4.02-3.98 (m, 2H), 3.96-3.93 (m, 1H), 3.89-3.86 (m, 1H), 3.74-3.70 (app dd, J_1 = 3.2, J_2 = 11.6, 1H), 3.66-3.62 (app dd, J_1 = 5.6, J_2 = 11.6, 1H), 3.47-3.43 (app dd, J_1 = 3.6, J_2 = 13.6, 1H), 3.37-3.32 (m, 1H) ppm. ^{13}C NMR (100 MHz, CD_3OD): δ, 164.0, 157.4, 154.3, 152.4, 126.1, 112.1, 108.3, 107.3, 98.2, 85.2, 83.1, 80.2, 78.8, 63.2, 51.5, 46.2 ppm.

7-((((2R,3S,4S,5R)-3,4-Dihydroxy-5-(hydroxymethyl)tetrahydrofuran-2-yl)methyl)amino)-4-((4-(3-fluoropropyl)-1H-1,2,3-triazol-1-yl)methyl)-2H-chromen-2-one (ManCou-F): Synthesis was carried out following ref.[4] The salt **4** (100 mg, 0.24 mmol, 1 eq.) was dissolved in acetonitrile/water 1:1 mixture (*v/v*, up to 4 mL) and the solution was purged with Ar for few minutes. 5-fluoro-1-pentyne (5 eq.) was added, followed by CuI (2 eq.), sodium L-ascorbate (15 eq.), and DIEA (15 eq.). The headspace of the vial was purged with Ar for 1 minute, and the resulting suspension was stirred vigorously at room temperature for 2 h. The mixtures were then concentrated to dryness under reduced pressure, absorbed on silica, and purified by column chromatography on silica gel using 0−40% methanol in CH_2Cl_2 mixtures. No attempts were made to optimize the yields of the products. HRMS (ESI): m/z $[M + H]^+$ calc'd for $C_{21}H_{26}FN_4O_6$: 449.18367; found: 449.18286. ^{1}H NMR (400 MHz, CD_3OD): δ, 7.90 (s, 1H), 7.48-7.46 (d, J = 8.8, 1H), 6.68-6.65 (dd, J_1 = 2.4, J_2 = 8.8, 1H), 6.49 (d, J = 2.4, 1H), 5.76 (bs, 2H), 5.42 (s, 1H), 4.54-4.39 (dt, J_1 = 5.8, J_2 = 47.2, 2H), 4.02-3.98 (m, 2H), 3.95-3.93 (m, 1H), 3.89-3.85 (m, 1H), 3.73-3.69 (app dd, J_1 = 3.6, J_2 = 11.6, 1H), 3.66-3.61 (app dd, J_1 = 5.6, J_2 = 11.6, 1H), 3.46-3.42 (app dd, J_1 = 3.6, J_2 = 13.6, 1H), 3.36-3.30 (m, 1H), 2.87-2.83 (t, J = 8.0, 2H), 2.12-1.99 (m, 2H) ppm. ^{13}C NMR (100 MHz, CD_3OD): δ, 163.6 (Cq), 157.4 (Cq), 154.5 (Cq), 152.1 (Cq), 148.8 (Cq), 125.9, 124.4, 112.2, 108.0 (Cq), 107.3, 98.2, 85.3, 84.7-83.0 (d, J = 162, CH_2), 83.1, 80.2, 78.8, 63.2 (CH_2), 50.9 (CH_2), 46.2 (CH_2), 31.2 (d, J = 19.8, CH_2), 22.2 (d, J = 6.1, CH_2) ppm. ^{19}F NMR (376 MHz, CD_3OD): δ, -222.4 (m, 1F) ppm.

4. HRMS, ^{1}H and ^{13}C NMR spectra

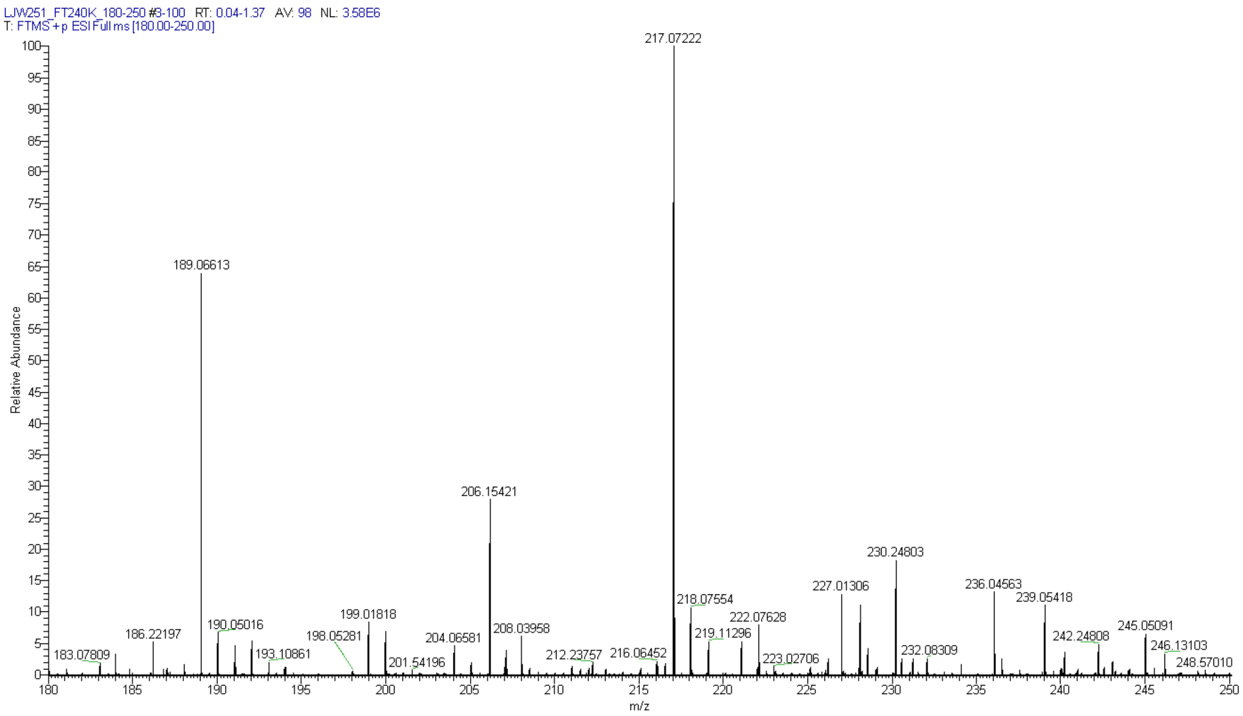

Figure S8. High-resolution mass spectrum of compound **3**

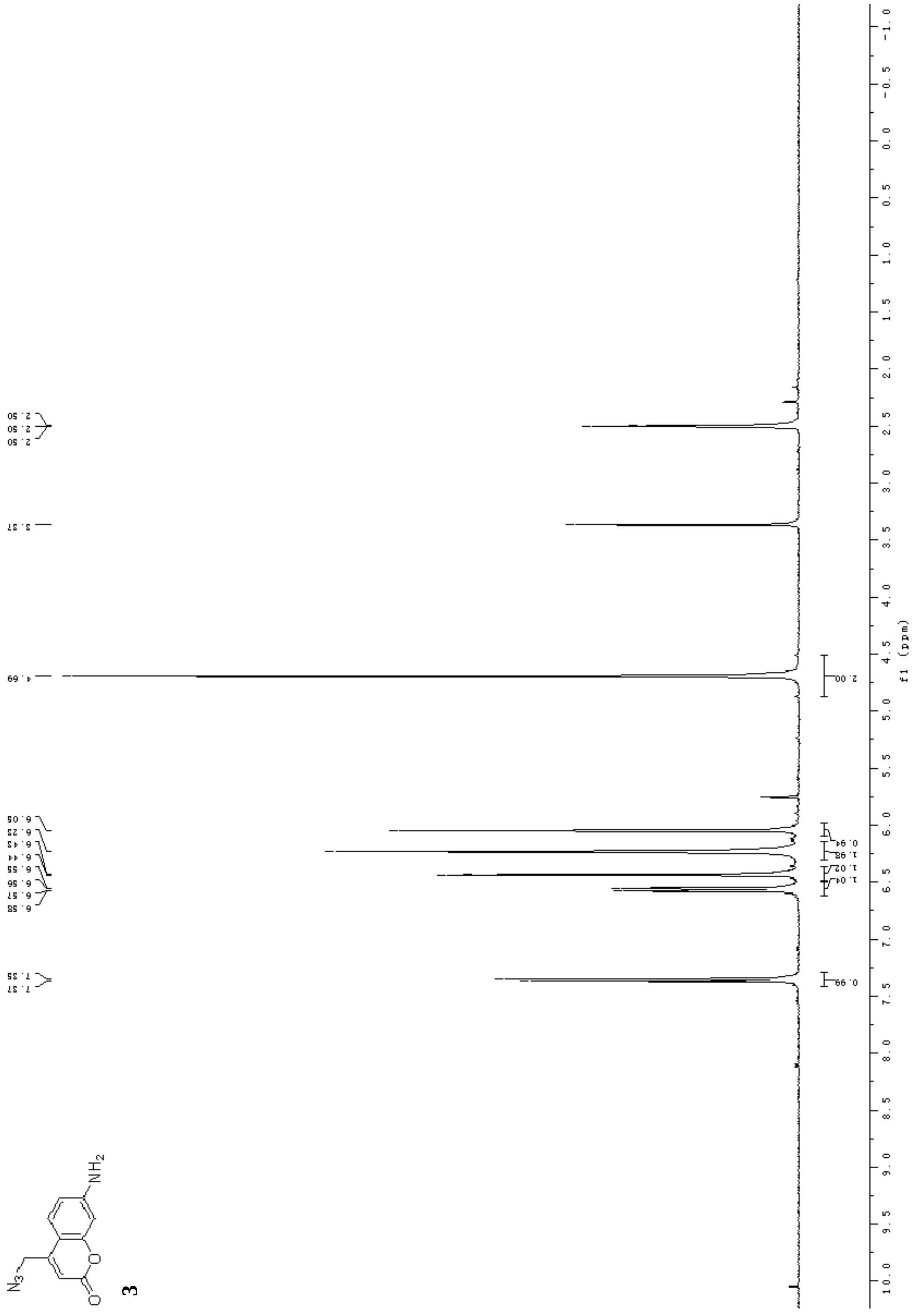

Figure S9. ^{1}H NMR spectrum of **3** (DMSO-d$_6$, 400 MHz).

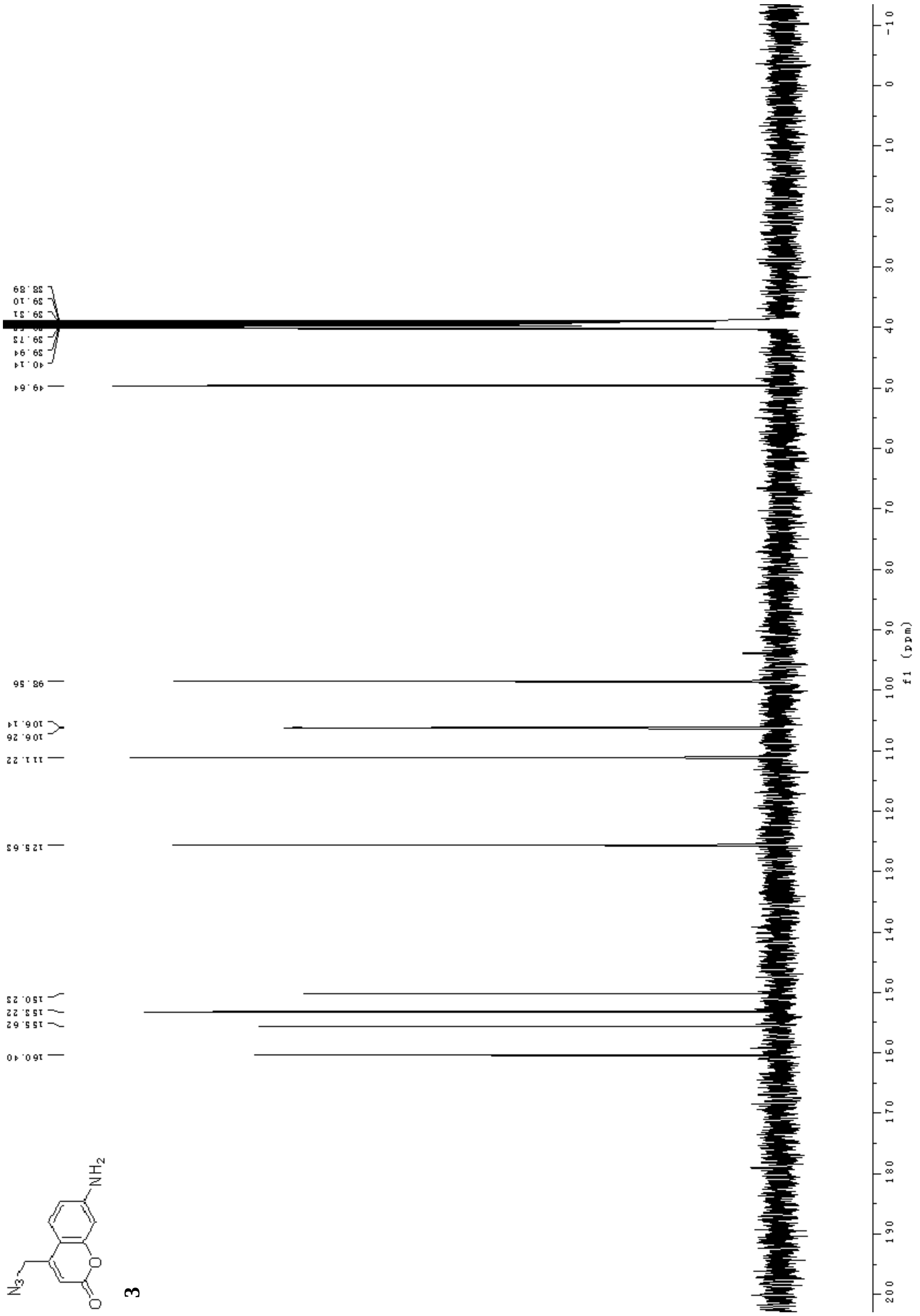

Figure S10. ^{13}C NMR spectrum of **3** (DMSO-d$_6$, 100 MHz).

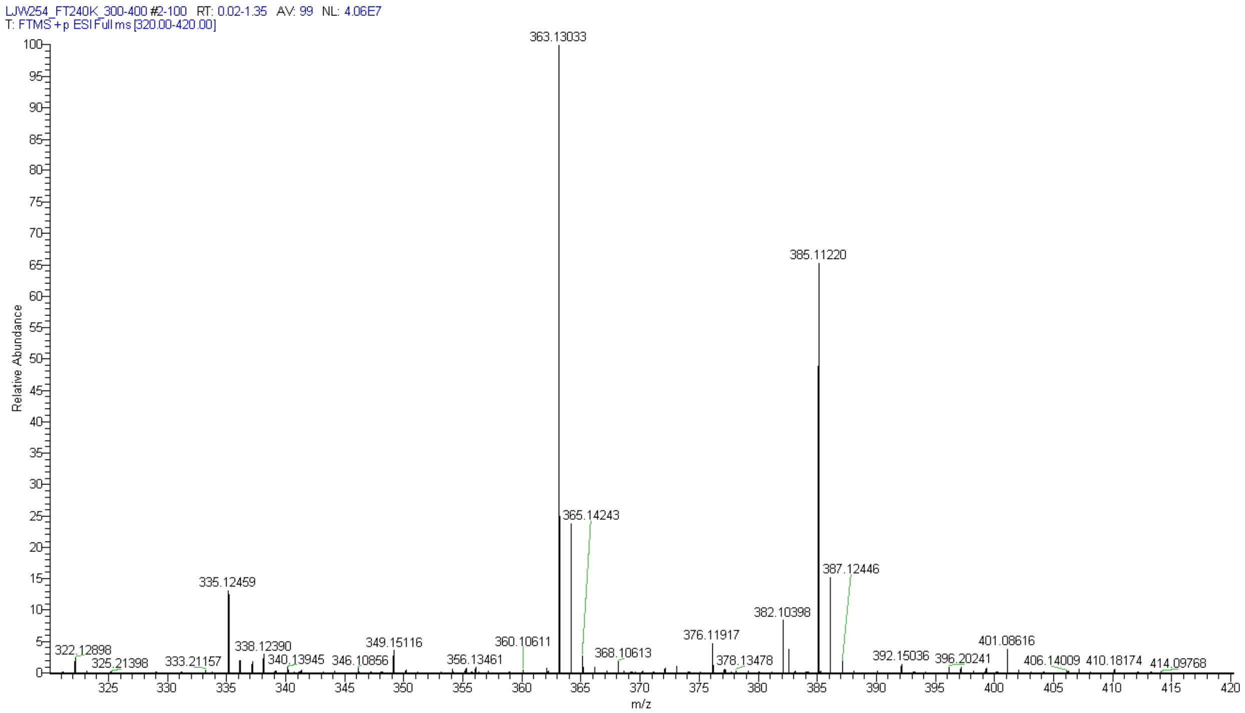

Figure S11. High-resolution mass spectrum of compound **4**

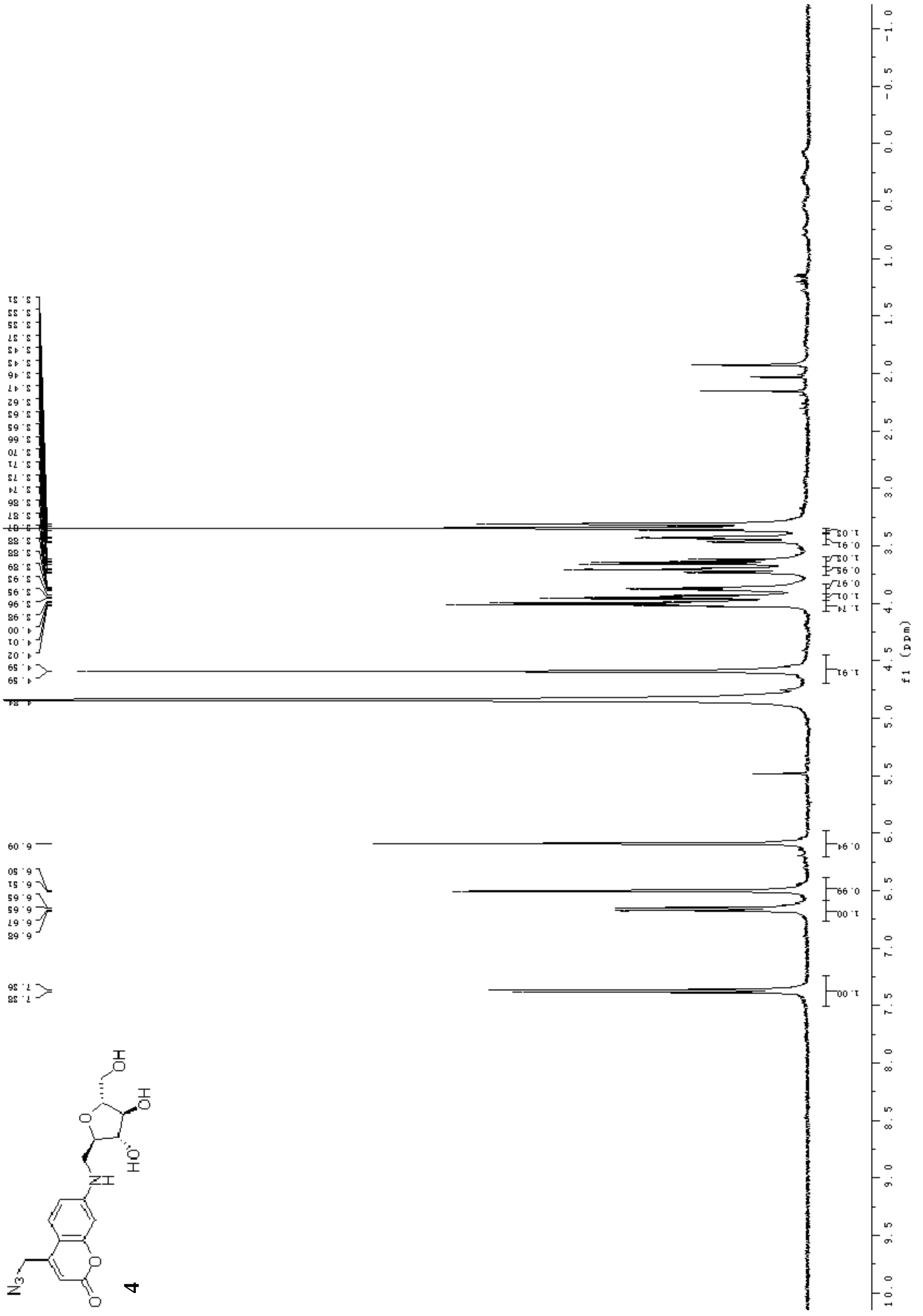

Figure S12. ^{1}H NMR spectrum of **4** (CD$_3$OD, 400 MHz).

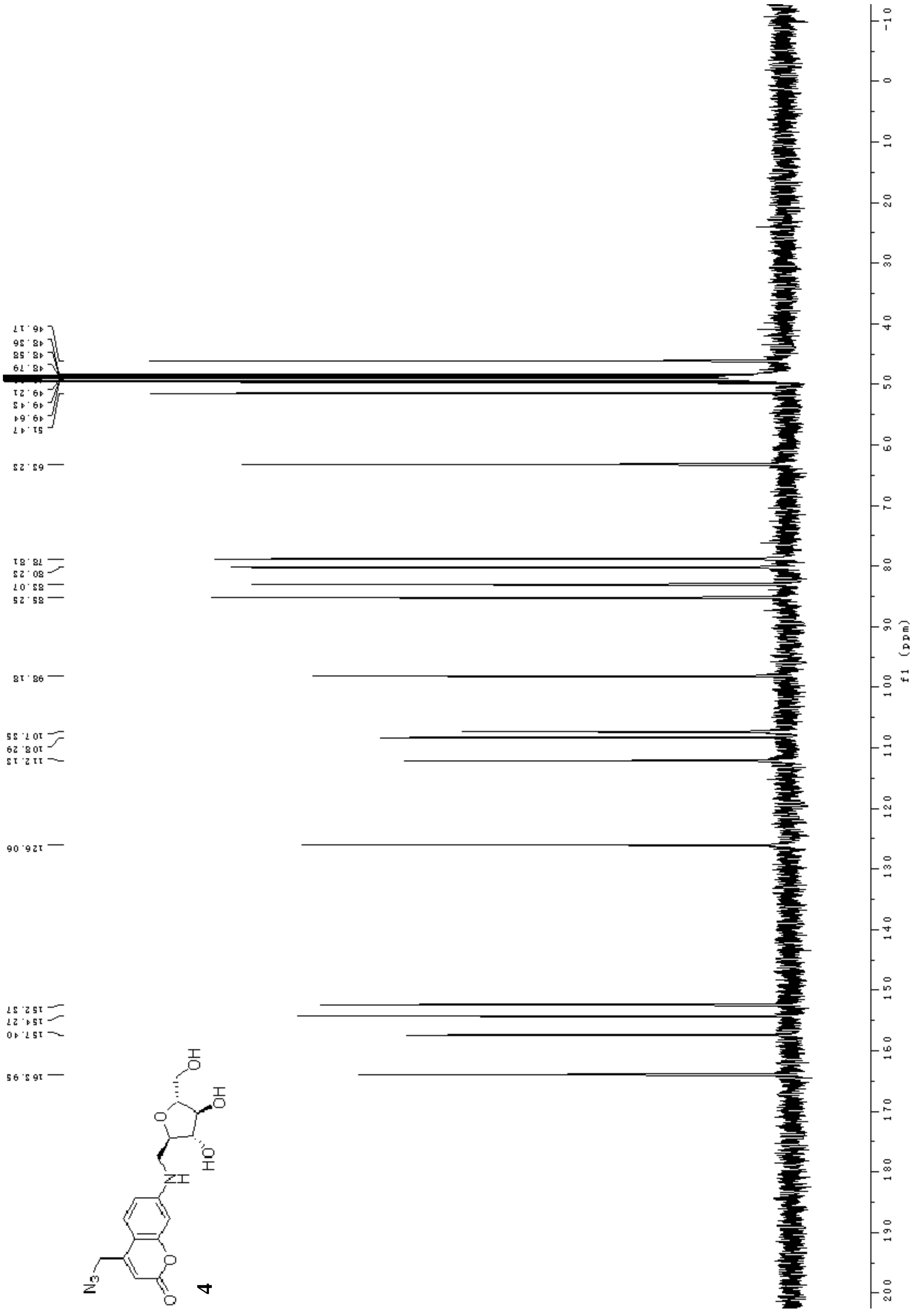

Figure S13. ^{13}C NMR spectrum of **4** (CD_3OD, 100 MHz).

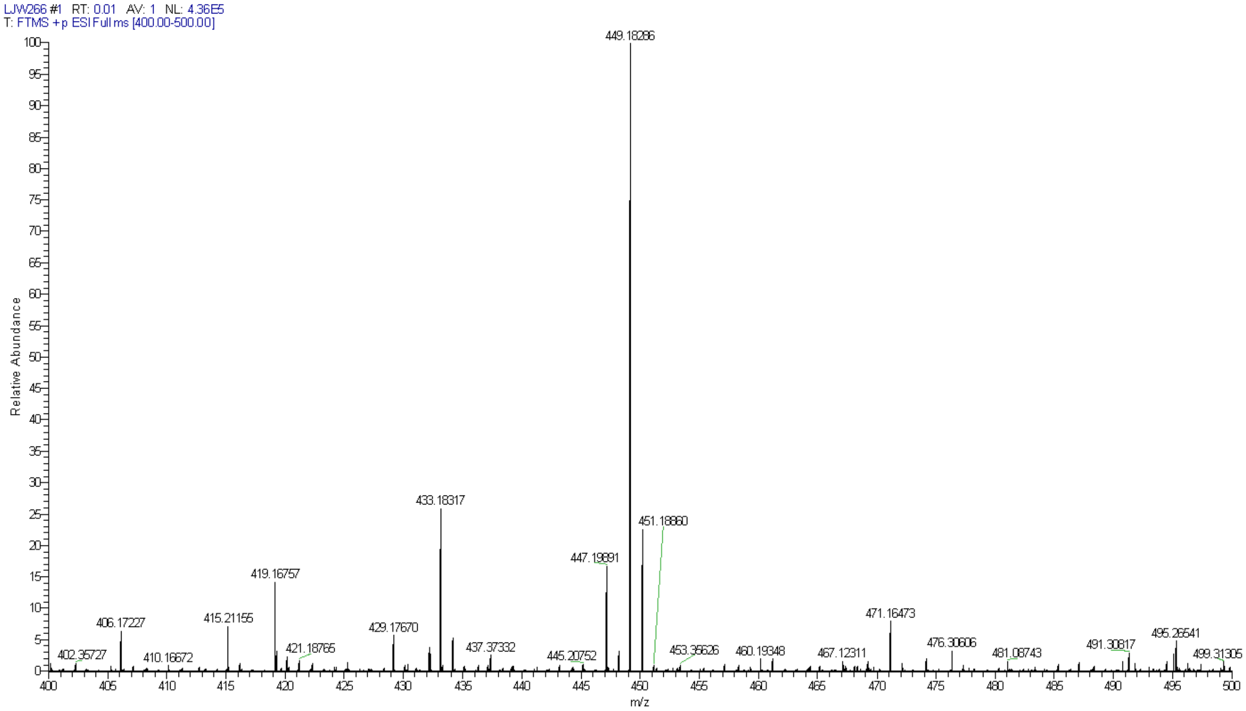

Figure S14. High-resolution mass spectrum of **ManCou-F**

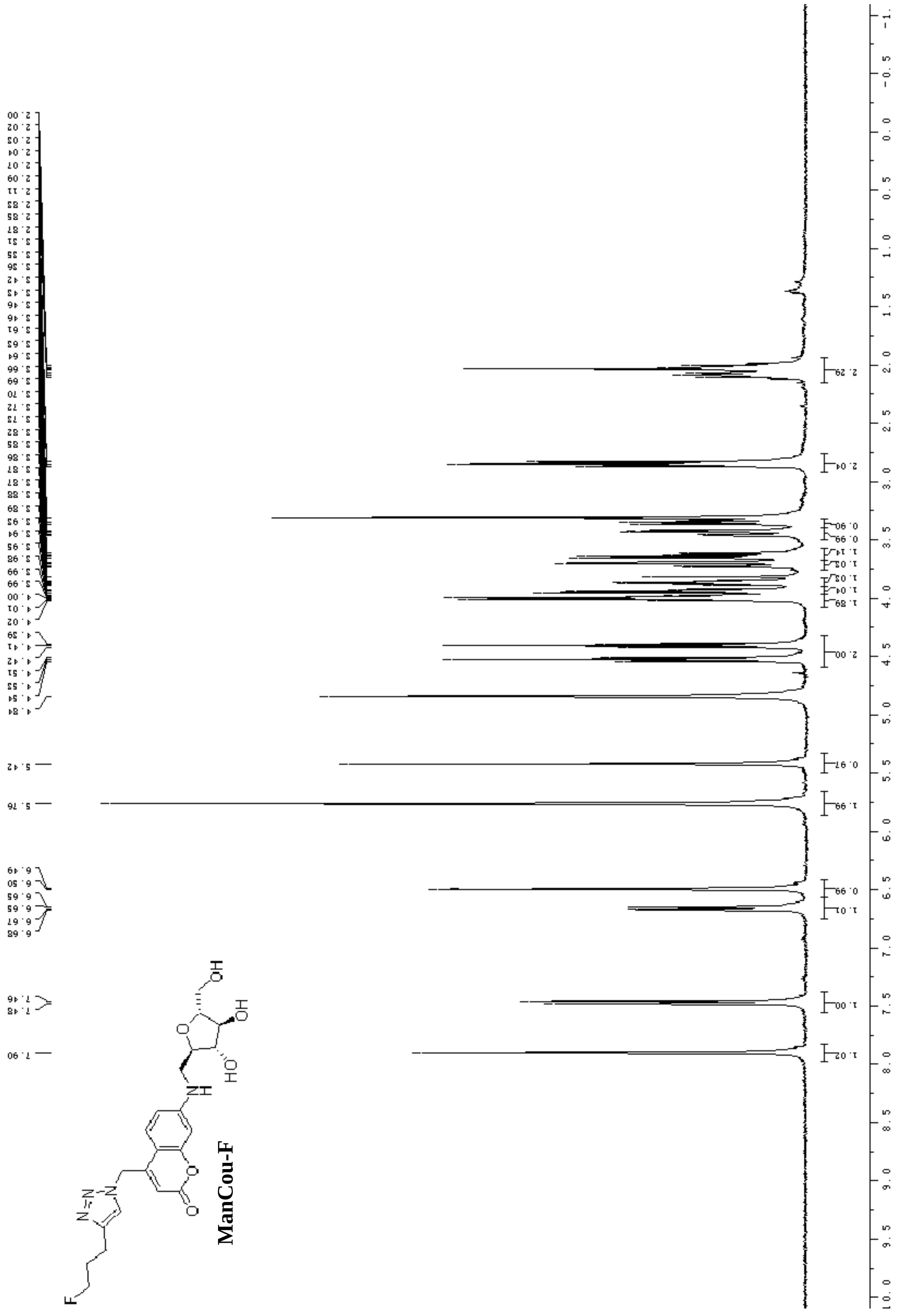

Figure S15. ^{1}H NMR spectrum of **ManCou-F** (CD_3OD, 400 MHz).

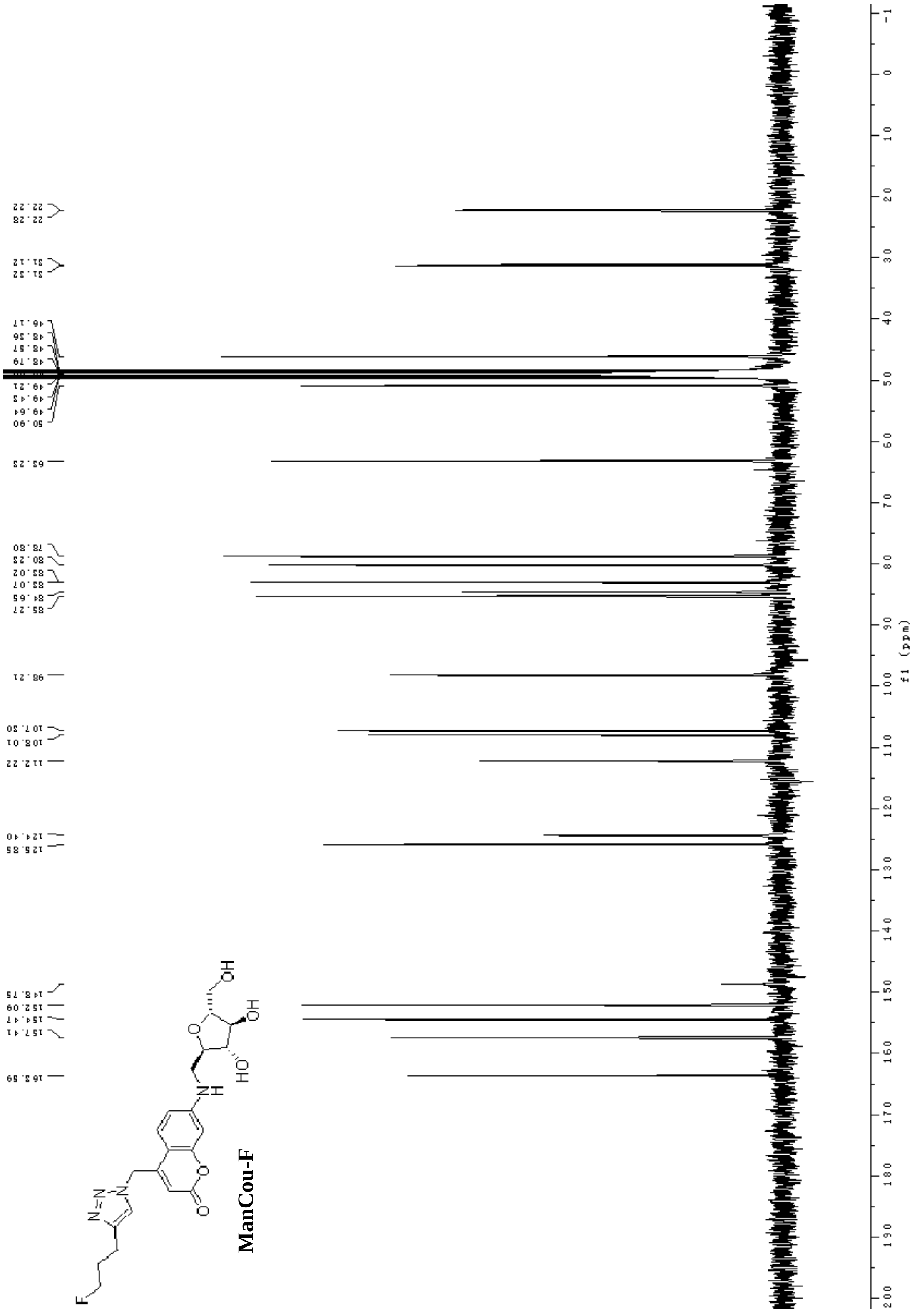

Figure S16. ^{13}C NMR spectrum of **ManCou-F** (CD_3OD, 100 MHz).

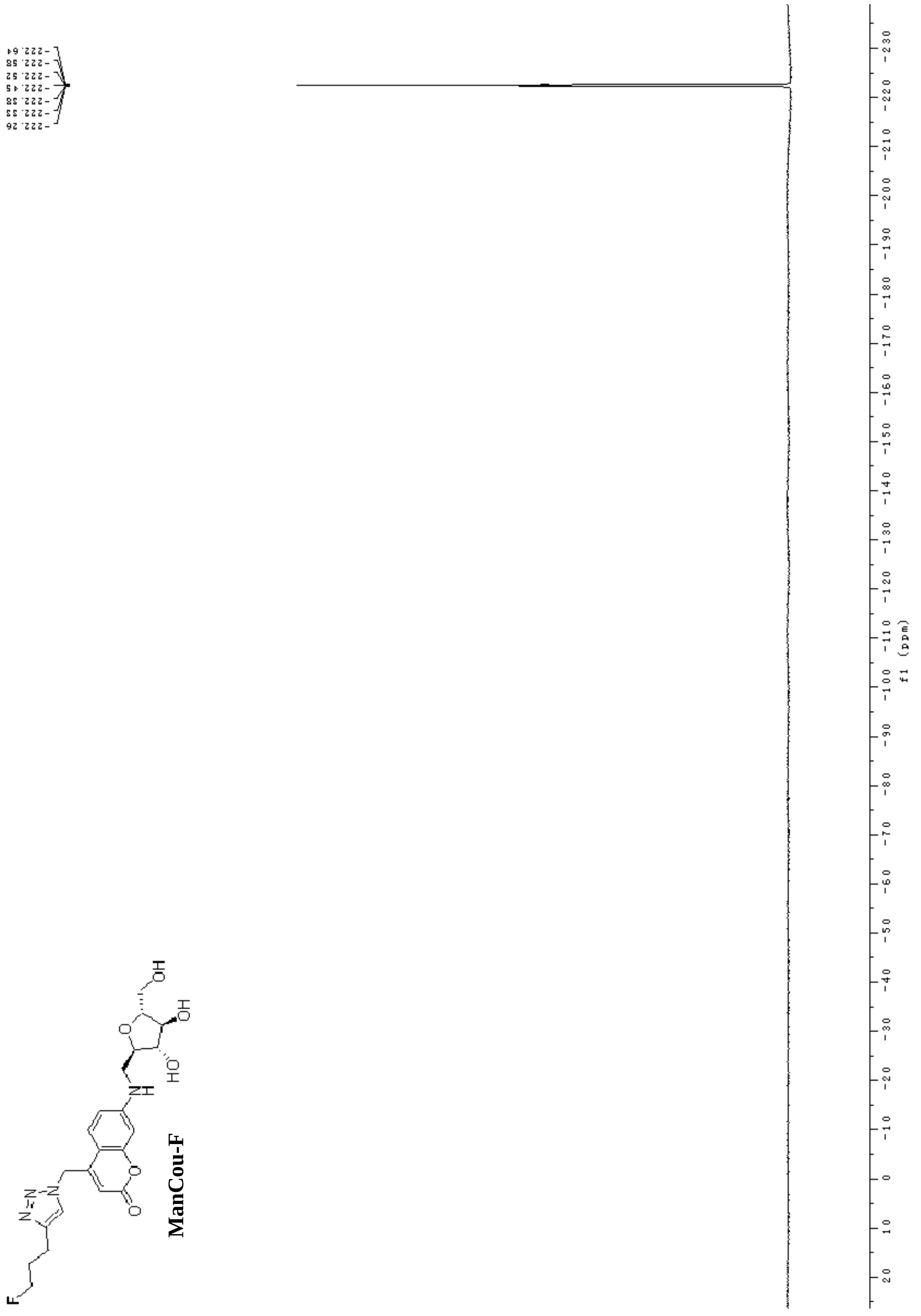

Figure S17. ^{19}F NMR spectrum of **ManCou-F** (CD$_3$OD, 376 MHz).

5. References

1. Oshikawa, Y.; Ojida, A., PET-dependent fluorescence sensing of enzyme reactions using the large and tunable pKa shift of aliphatic amines. *Chem. Commun.* **2013,** *49* (97), 11373-5.
2. Begoyan, V. V.; Weselinski, L. J.; Xia, S.; Fedie, J.; Kannan, S.; Ferrier, A.; Rao, S.; Tanasova, M., Multicolor GLUT5-permeable fluorescent probes for fructose transport analysis. *Chem. Commun.* **2018,** *54* (31), 3855-3858.
3. Hausner, S. H.; Marik, J.; Gagnon, M. K.; Sutcliffe, J. L., In vivo positron emission tomography (PET) imaging with an alphavbeta6 specific peptide radiolabeled using 18F-"click" chemistry: evaluation and comparison with the corresponding 4-[18F]fluorobenzoyl- and 2-[18F]fluoropropionyl-peptides. *J. Med. Chem.* **2008,** *51* (19), 5901-4.
4. van Wilderen, L. J.; Neumann, C.; Rodrigues-Correia, A.; Kern-Michler, D.; Mielke, N.; Reinfelds, M.; Heckel, A.; Bredenbeck, J., Picosecond activation of the DEACM photocage unravelled by VIS-pump-IR-probe spectroscopy. *Phys. Chem. Chem. Phys.* **2017,** *19* (9), 6487-6496.
